# New insights into the role of plasmids from probiotic *Lactobacillus pentosus* MP-10 in Aloreña table olive brine fermentation

**DOI:** 10.1038/s41598-019-47384-1

**Published:** 2019-07-29

**Authors:** Hikmate Abriouel, Beatriz Pérez Montoro, Juan José de la Fuente Ordoñez, Leyre Lavilla Lerma, Charles W. Knapp, Nabil Benomar

**Affiliations:** 1 Área de Microbiología, Departamento de Ciencias de la Salud, Facultad de Ciencias Experimentales, Universidad de Jaén, 23071 Jaén, Spain; 2 Centre of Water, Environment, Sustainability and Public Health; Department of Civil and Environmental Engineering, University of Strathclyde, Glasgow, Scotland United Kingdom

**Keywords:** Applied microbiology, Microbial genetics

## Abstract

*In silico* analysis of *Lactobacillus pentosus* MP-10 plasmids (pLPE-1 to pLPE-5) suggests that plasmid-borne genes mediate the persistence of lactobacilli during olive fermentation and enhance their probiotic properties and their competitiveness in several ecological niches. The role of plasmids in the probiotic activities of *L. pentosus* MP-10 was investigated by plasmid-curing process which showed that plasmids contribute in increased metal tolerance and the biosequestration of several metals such as iron, aluminium, cobalt, copper, zinc, cadmium and mercury. Statistically significant differences in mucin adhesion were detected between the uncured and the cured *L. pentosus* MP-10, which possibly relied on a serine-rich adhesin (*sraP*) gene detected on the pLPE-2 plasmid. However, plasmid curing did not affect their tolerance to gastro-intestinal conditions, neither their growth ability under pre-determined conditions, nor auto-aggregation and pathogen co-aggregation were changed among the cured and uncured *L. pentosus* MP-10. These findings suggest that *L. pentosus* MP-10 plasmids play an important role in gastro-intestinal protection due to their attachment to mucin and, thus, preventing several diseases. Furthermore, *L. pentosus* MP-10 could be used as a bioquencher of metals in the gut, reducing the amount of these potentially toxic elements in humans and animals, food matrices, and environmental bioremediation.

## Introduction

Table olive fermentation is the oldest practice by our ancestors to preserve vegetables and to also produce different flavours and textures. Additionally, fermented table olives remain an important economy for many production countries and a component of the Mediterranean diet (and recommended as part of the Healthy Eating Pyramid published in 2010, https://dietamediterranea.com/). The high nutritional value of fermented table olives (e.g., their content of carbohydrates, fiber, minerals, vitamins, fatty acids, and amino acids) and their role as potential source of probiotic lactobacilli of vegetable origin^1–5^ make them very attractive from an economic and social point of view. *Lactobacillus* genus is the most representative and heterogeneous member of lactic acid bacteria (LAB) group currently consisting of 237 species (as of December 2018 in www.bacterio.net) since they harbour in their genome a plethora of genes involved with a wide array of functional properties^6,7^. *Lactobacillus* spp. are principal bacteria in olive fermentation processes, possessing many biochemical and physiological traits to ferment several carbohydrates and tolerate stress^8^. These phenotypes are important as the brine environment represent harsh conditions for bacterial growth with low nutrient availability, saltiness, low pH and the presence of antimicrobials (e.g., phenolic compunds and oleuropein); thus, highly robust *L. plantarum* and *L. pentosus* are frequently isolated from the end of olive fermentation^1,8,9^. Furthermore, Perpetuini, *et al*.^10^ demonstrated by transposon mutagenesis that the high capacity of *L. plantarum* and *L. pentosus* to survive in the hostile, brine environments was due to critical genes encoding proteins involved in carbohydrate metabolism, membrane structure and function, and gene-expression regulation. They further suggested that the *obaD* gene, which encodes a putative membrane protein strictly specific to *L. pentosus/L. plantarum* species, may be one of the key elements involved in their efficient adaptation to several conditions in many fermented food processes and natural ecosystems^10^.

Aloreña green table olive fermentation is a spontaneous process relying on *L. pentosus* strains and yeasts^1,9^. Resistance, persistence and predominance of *Lactobacillus* spp. in green table olive fermentation is due to their genetic variation and plasticity related to their chromosome and plasmids. In fact, *L. pentosus* species isolated from olive fermentation harbours the largest genome recognized to date and several plasmids (range: n = 5 to 7)^11–13^. However, *L. plantarum* contains the largest plasmids among the genus *Lactobacillus*^14,15^ such as *L. plantarum* 16, which harbors 10 plasmids ranging 6.46–74.08 kb^16^. Most of the *Lactobacillus* plasmids are cryptic; however, they possess important properties such as antibiotic resistance, exopolysaccharide production, antimicrobial activity, bacteriocin synthesis, bacteriophage resistance, carbohydrate metabolism, host colonization and probiotic activity^17–22^. On the other hand, megaplasmids were also detected in *Lactobacillus* sp., up to 490 kb^23^. In this study, we analyzed *in silico* five plasmids harboured by *L. pentosus* MP-10 isolated from naturally fermented Aloreña green table olives^2,9,12^. Moreover, we aimed to better understand the underlying functional and probiotic properties of these plasmids using curing plasmid experiments; in particular, we examined their physiological traits in metal tolerance and biosorption, antimicrobial activity and adaptation to gastro-intestinal conditions to determine possible probiotic applications of this bacterium.

## Results

### General features of *L. pentosus* MP-10 plasmids

We have already reported the sequencing of *L. pentosus* MP-10 genome^12^, which consisted of a single circular chromosome of 3,698 kbp and five plasmids ranging 29–46 kbp (accession numbers FLYG01000001 to FLYG01000006). Sequence annotation was done using the Prokka annotation pipeline, version 1.11^24^ as previously reported by Abriouel, *et al*.^12^. The general features of the circular five plasmids^2^ are reported in Table 1. The average GC content of *L. pentosus* MP-10 plasmids ranged 39.52–42.50%, slightly lower than the host chromosome (with GC value of 46.32%). Furthermore, the GC contents of *L. pentosus* MP-10 plasmids were among the highest of known *L. pentosus* plasmids. All open reading frames in *L. pentosus* MP-10 plasmids are greater than 34 amino acids (Tables 2–6). Blast search for homology revealed lower identity with other plasmids in the database; however depending on coverage percentage, some regions harboring several genes in *L. pentosus* MP-10 plasmids were highly related with plasmids of *Lactobacillus* species isolated from foods like fermented olives, kimchi, koumiss, tofu or raw sausages, and also from human saliva (Table 1).

**Table 1:** General features of circular plasmids from *L. pentosus* MP-10.

Plasmid	Size (bp)	G + C (%)	CDs	Hypothetical proteins	Similarity to plasmids (BlastN)	
					Identity in *Lactobacillus* (Isolation source)	Coverage (%)
pLPE-1	29,077	40.77	35	20	92% in *L. plantarum* subsp. *plantarum* TS12 plasmid pLP12-4 (“Stinky tofu”)	59
pLPE-2	34,764	39.93	36	13	99% in *L. pentosus* IG1, annotated genomic scaffold00003 (Spanish-style green-olive fermentations)	47
pLPE-3	38,717	42.50	42	10	91% in *L. plantarum* strain BLS41 plasmid pLPBLS41_3 (Kimchi)	28
pLPE-4	43,946	40.09	53	32	91% in *L. casei* str. Zhang plasmid plca36 (Koumiss)	75
pLPE-5	46,498	39.52	58	32	99% in *L. plantarum* WCFS1 1.25 plasmid pWCFS103 (Human saliva)	51

**Table 2:** Genes determined in pLPE-1 plasmid of *Lactobacillus pentosus* MP-10 isolated from naturally fermented Aloreña table olives.

Gene ID	Gene	Position	Strand	Gen length (bp)	Protein description	GO terms	Similarity to proteins in *Lactobacillus*
XX999_03518	*XX999_03518*	804–950	−	147	Hypothetical protein	—	98% identity in *L. paracasei* subsp. *paracasei* Lpp70
XX999_03519	*XX999_03519*	963–1271	−	309	Phage integrase family protein	—	87% identity in *Lactobacillus*
XX999_03520	*XX999_03520*	1238–1651	−	414	Hypothetical protein	—	99% identity in *L. plantarum* IPLA88
XX999_03521	*XX999_03521*	1871–2215	+	345	Toxin MazF	DNA binding (MF); RNA binding (MF); endoribonuclease activity (MF); endoribonuclease activity, producing 5′-phosphomonoesters (MF); negative regulation of cell growth (BP); regulation of mRNA stability (BP); RNA phosphodiester bond hydrolysis, endonucleolytic (BP)	100% identity in *L. pentosus*
XX999_03522	*XX999_03522*	2675–3739	−	1065	Hypothetical protein	—	99% identity in *L. xiangfangensis*
XX999_03523	*XX999_03523*	3901–4380	−	480	Hypothetical protein	—	100% identity in *L. pentosus*
XX999_03524	*XX999_03524*	4989–5576	−	588	Initiator Replication protein	—	98% identity in *L. plantarum*
XX999_03525	*XX999_03525*	6296–6490	−	195	Hypothetical protein	—	100% identity in *L. pentosus* IG1
XX999_03526	*mobA_4*	7058–8221	+	1164	Mobilization protein A	Conjugation (BP); DNA binding (MF); DNA-directed RNA polymerase activity (MF); DNA topoisomerase type I activity (MF); cytoplasm (CC); metal ion binding (MF)	100% identity in *L. pentosus*
XX999_03527	*XX999_03527*	8218–8910	+	693	Hypothetical protein	—	100% identity in *L. pentosus*
XX999_03528	*XX999_03528*	9111–9866	−	756	Initiator Replication protein	—	100% identity in *L. plantarum* IPLA88
XX999_03529	*XX999_03529*	10508–10957	+	450	Hypothetical protein	—	100% identity in *L. pentosus*
XX999_03530	*XX999_03530*	10954–11157	+	204	Hypothetical protein	—	100% identity in *L. pentosus*
XX999_03531	*XX999_03531*	11306–11668	−	363	Hypothetical protein	—	100% identity in *L. pentosus*
XX999_03532	*XX999_03532*	11912–12271	−	360	Hypothetical protein	−	99% identity in *L. brevis*
XX999_03533	*XX999_03533*	12284–12871	−	588	Site-specific tyrosine recombinase XerC	—	99% identity in *L. plantarum* 2025
XX999_03534	*XX999_03534*	12949–13212	+	264	Putative regulator PrlF	Regulation of cell growth (BP); DNA binding (MF); sequence-specific DNA binding transcription factor activity (MF); cytoplasm (CC); transcription, DNA-templated (BP); enzyme binding (MF); negative regulation of transcription, DNA-templated (BP)	100% identity in *L. plantarum*
XX999_03535	*ndoA_2*	13212–13559	+	348	mRNA interferase EndoA	DNA binding (MF); RNA binding (MF); endoribonuclease activity (MF); endoribonuclease activity, producing 5′-phosphomonoesters (MF); negative regulation of cell growth (BP); regulation of mRNA stability (BP); RNA phosphodiester bond hydrolysis, endonucleolytic (BP)	98% identity in *Lactobacillus*
XX999_03536	*XX999_03536*	14021–15085	−	1065	Hypothetical protein	—	99% identity in *L. xiangfangensis*
XX999_03537	*XX999_03537*	15164–15751	−	588	Hypothetical protein	—	100% identity in *L. pentosus*
XX999_03538	*XX999_03538*	15993–16928	−	936	Initiator Replication protein	—	99% identity in *L. plantarum* subsp. *plantarum*
XX999_03539	*XX999_03539*	17648–17842	−	195	Hypothetical protein	—	100% identity in *L. pentosus* IG1
XX999_03540	*mobA_5*	18410–19573	+	1164	Mobilization protein A	Conjugation (BP); DNA binding (MF); DNA-directed RNA polymerase activity (MF); DNA topoisomerase type I activity (MF); cytoplasm (CC); metal ion binding (MF)	95% identity in *L. plantarum*
XX999_03541	*XX999_03541*	19570–20262	+	693	Hypothetical protein	—	98% identity in *L. plantarum* 2025
XX999_03542	*XX999_03542*	20463–21218	−	756	Initiator Replication protein	—	100% identity in *L. plantarum* IPLA88
XX999_03543	*XX999_03543*	21860–22309	+	450	Hypothetical protein	—	100% identity in *L. pentosus*
XX999_03544	*XX999_03544*	22306–22509	+	204	Hypothetical protein	—	100% identity in *L. pentosus*
XX999_03545	*XX999_03545*	22658–23020	−	363	Hypothetical protein	—	100% identity in *L. pentosus*
XX999_03546	*XX999_03546*	23264–23623	−	360	Hypothetical protein	—	100% identity in *L. pentosus*
XX999_03547	*XX999_03547*	23636–24223	−	588	Site-specific tyrosine recombinase XerC	—	99% identity in *L. plantarum* 2025
XX999_03548	*XX999_03548*	24300–24563	+	264	Putative regulator PrlF	Regulation of cell growth (BP); DNA binding (MF); sequence-specific DNA binding transcription factor activity (MF); cytoplasm (CC); transcription, DNA-templated (BP); enzyme binding (MF); negative regulation of transcription, DNA-templated (BP)	100% identity in *L. plantarum* 2025
XX999_03549	*ndoA_3*	24563–24910	+	348	mRNA interferase EndoA	DNA binding (MF); RNA binding (MF); endoribonuclease activity (MF); endoribonuclease activity, producing 5′-phosphomonoesters (MF); negative regulation of cell growth (BP); regulation of mRNA stability (BP); RNA phosphodiester bond hydrolysis, endonucleolytic (BP)	98% identity in *Lactobacillus*
XX999_03550	*XX999_03550*	25372–26436	−	1065	Hypothetical protein	—	99% identity in *L. xiangfangensis*
XX999_03551	*XX999_03551*	26515–27102	−	588	Hypothetical protein	—	100% identity in *L. pentosus*
XX999_03552	*XX999_03552*	27344–28279	−	936	Initiator Replication protein	—	99% identity in *L. plantarum* subsp. *plantarum*

BP, biological process; CC, celular component; MF, molecular function.

**Tablze 3:** Genes determined in pLPE-2 plasmid of *Lactobacillus pentosus* MP-10 isolated from naturally fermented Aloreña table olives.

Gene ID	Gene	Position	Strand	Gen length (bp)	Protein description	GO terms	Similarity to proteins in *Lactobacillus*
XX999_03611	*clcA_2*	189–1568	+	1380	H(+)/Cl(−) exchange transporter ClcA	Voltage-gated chloride channel activity (MF); integral component of plasma membrane (CC); antiporter activity (MF)	99% identity in *L. pentosus* SLC13 plasmid pSLC13
XX999_03612	*XX999_03612*	2377–3297	+	921	Integrase core domain protein	—	99% identity in *L. pentosus*
XX999_03613	*XX999_03613*	4093–4353	−	261	Phd_YefM	DNA binding (MF); transcription, DNA-templated (BP); regulation of transcription, DNA-templated (BP)	100% identity in *L. plantarum* CMPG5300
XX999_03614	*XX999_03614*	4535–5902	−	1368	Transposase DDE domain protein	—	82% identity in *L. backii*
XX999_03615	*sraP*	6332–8032	+	1701	Serine-rich adhesin for platelets precursor	Calcium ion binding (MF); extracellular region (CC); cell wall (CC); pathogenesis (BP); membrane (CC)	60% identity in *L. plantarum* O2T60C
XX999_03616	*yusO*	8090–8527	+	438	Putative HTH-type transcriptional regulator YusO	DNA binding (MF); sequence-specific DNA binding transcription factor activity (MF); intracellular (CC); transcription initiation from RNA polymerase II promoter (BP)	99% identity in *L. pentosus* IG1
XX999_03617	*XX999_03617*	8891–9229	+	339	Hypothetical protein	—	100% identity in *L. plantarum* plasmid pLP9000_06
XX999_03618	*XX999_03618*	9187–9690	+	504	Transposase DDE domain protein	—	100% identity in *L. plantarum* UCMA 3037
XX999_03619	*XX999_03619*	9650–9883	−	234	Hypothetical protein	—	62% identity in *L. plantarum* subsp. *plantarum*
XX999_03620	*XX999_03620*	10191–11729	−	1539	Hypothetical protein	—	—
XX999_03621	*soj_3*	12508–13425	+	918	Sporulation initiation inhibitor protein Soj	ATP binding (MF); oxidoreductase activity (MF); hydrolase activity (MF); sporulation resulting in formation of a cellular spore (BP); negative regulation of sporulation resulting in formation of a cellular spore (BP)	99% identity in *L. pentosus* DSM 20314
XX999_03622	*XX999_03622*	13409–13696	+	288	Hypothetical protein	—	100% identity in *L. plantarum*
XX999_03623	*XX999_03623*	13862–15037	+	1176	Transposase, Mutator family	—	100% identity in *L. pentosus*
XX999_03624	*XX999_03624*	15547–15732	−	186	Hypothetical protein	—	53% identity in *L. plantarum*
XX999_03625	*XX999_03625*	16181–16477	−	297	Hypothetical protein	—	100% identity in *L. pentosus* DSM 20314
XX999_03626	*XX999_03626*	16698–17015	+	318	Hypothetical protein	—	100% identity in *L. plantarum*
XX999_03627	*XX999_03627*	17186–17482	+	297	Transposase DDE domain protein	—	100% identity in *L. plantarum*
XX999_03628	*XX999_03628*	17582–17986	+	405	D-alanine/D-serine/glycine permease	—	99% identity in *L. plantarum*
XX999_03629	*XX999_03629*	18547–18774	−	228	Hypothetical protein	—	100% identity in *L. pentosus* DSM 20314
XX999_03630	*soj_4*	19677–20477	+	801	Chromosome-partitioning ATPase Soj	ATP binding (MF)	100% identity in *L. pentosus* IG1
XX999_03631	*XX999_03631*	20479–20826	+	348	Hypothetical protein	—	100% identity in *L. plantarum* CMPG5300
XX999_03632	*XX999_03632*	21412–22314	−	903	Hypothetical protein	—	100% identity in *L. pentosus* IG1
XX999_03633	*bin3_4*	22401–23033	−	633	Putative transposon Tn552 DNA-invertase bin3	Recombinase activity (MF); DNA binding (MF); DNA integration (BP); transposition (BP)	99% identity in *L. plantarum* 16
XX999_03634	*XX999_03634*	23329–23958	+	630	Integrase core domain protein	—	100% identity in *L. pentosus* IG1
XX999_03635	*nrdF2_2*	24087–25037	−	951	Ribonucleoside-diphosphate reductase subunit beta nrdF2	Ribonucleoside-diphosphate reductase activity, thioredoxin disulfide as acceptor (MF); ribonucleoside-diphosphate reductase complex (CC); DNA replication (BP); deoxyribonucleoside diphosphate metabolic process (BP); deoxyribonucleotide biosynthetic process (BP); metal ion binding (MF)	100% identity in *L. pentosus* IG1
XX999_03636	*nrdF*	25052–25978	−	927	Ribonucleoside-diphosphate reductase 2 subunit beta	Ribonucleoside-diphosphate reductase activity, thioredoxin disulfide as acceptor (MF); ribonucleoside-diphosphate reductase complex (CC); DNA replication (BP); deoxyribonucleoside diphosphate metabolic process (BP); deoxyribonucleotide biosynthetic process (BP); metal ion binding (MF)	100% identity in *L. pentosus* IG1
XX999_03637	*nrdE*	26085–28253	−	2169	Ribonucleoside-diphosphate reductase 2 subunit alpha	Ribonucleoside-diphosphate reductase activity, thioredoxin disulfide as acceptor (MF); ATP binding (MF); DNA replication (BP)	100% identity in *L. pentosus* DSM 20314
XX999_03638	*XX999_03638*	28260–28697	−	438	Putative NrdI-like protein	—	100% identity in *L. plantarum* AY01
XX999_03639	*XX999_03639*	29395–29496	−	102	Hypothetical protein	—	100% identity in *L. plantarum* 2165
XX999_03640	*XX999_03640*	29486–29845	−	360	Putative hydrolase	—	99% identity in *L. plantarum* 2165
XX999_03641	*XX999_03641*	30683–30943	−	261	Hypothetical protein	Recombinase activity (MF); DNA binding (MF); DNA integration (BP)	100% identity in *L. plantarum* AY01
XX999_03642	*XX999_03642*	30999–31250	+	252	Transposase	—	100% identity in *L. pentosus*
XX999_03643	*XX999_03643*	31304–32146	+	843	Integrase core domain protein	—	99% identity in *L. plantarum*
XX999_03644	*XX999_03644*	32416–32805	−	390	Integrase core domain protein	—	99% identity in *L. plantarum*
XX999_03645	*XX999_03645*	32896–33381	−	486	Hypothetical protein	—	100% identity in *L. plantarum*
XX999_03646	*nhaS3_4*	33487–34641	+	1155	High-affinity Na(+)/H(+) antiporter NhaS3	Plasma membrane (CC); sodium ion transmembrane transporter activity (MF); antiporter activity (MF); solute:proton antiporter activity (MF); integral component of membrane (CC); sodium ion transmembrane transport (BP)	100% identity in *L. pentosus* IG1

BP, biological process; CC, celular component; MF, molecular function.

**Table 4:** Genes determined in pLPE-3 plasmid of *Lactobacillus pentosus* MP-10 isolated from naturally fermented Aloreña table olives.

Gene ID	Gene	Position	Strand	Gen length (bp)	Protein description	GO terms
XX999_00053	*XX999_00053*	146–412	−	267	Zeta toxin	—
XX999_00054	*XX999_00054*	586–783	−	198	Hypothetical protein	—
XX999_00055	*XX999_00055*	1002–1931	+	930	Integrase core domain protein	—
XX999_00056	*XX999_00056*	1934–2152	+	219	Hypothetical protein	—
XX999_00057	*soj_1*	3395–4204	+	810	Chromosome-partitioning ATPase Soj	DNA binding (MF); ATP binding (MF); chromosome segregation (BP); hydrolase activity (MF)
XX999_00058	*XX999_00058*	4197–4532	+	336	Hypothetical protein	—
XX999_00059	*XX999_00059*	4598–4771	+	174	Hypothetical protein	—
XX999_00060	*XX999_00060*	5611–6453	−	843	Integrase core domain protein	—
XX999_00061	*XX999_00061*	6507–6758	−	252	Transposase	—
XX999_00062	*XX999_00062*	6826–7092	−	267	Divergent AAA domain protein	—
XX999_00063	*ilvE_1*	7372–8394	−	1023	Putative branched-chain-amino-acid aminotransferase	Isoleucine biosynthetic process (BP); leucine biosynthetic process (BP); valine biosynthetic process (BP); L-leucine transaminase activity (MF); L-valine transaminase activity (MF); L-isoleucine transaminase activity (MF)
XX999_00064	*panE_1*	8444–9463	−	1020	2-dehydropantoate 2-reductase	Cytoplasm (CC); 2-dehydropantoate 2-reductase activity (MF); pantothenate biosynthetic process from valine (BP); NADP binding (MF)
XX999_00065	*yvdD_1*	9990–10559	−	570	LOG family protein YvdD	—
XX999_00066	*XX999_00066*	10970–11968	+	999	Integrase core domain protein	—
XX999_00067	*panE_2*	12688–13698	+	1011	2-dehydropantoate 2-reductase	Cytoplasm (CC); 2-dehydropantoate 2-reductase activity (MF); pantothenate biosynthetic process from valine (BP); NADP binding (MF)
XX999_00068	*XX999_00068*	13686–14087	−	402	Prephenate dehydratase	—
XX999_00069	*XX999_00069*	14032–14613	−	582	Transposase, Mutator family	—
XX999_00070	*asnB_1*	14954–16543	−	1590	Asparagine synthetase B [glutamine-hydrolyzing]	Asparagine synthase (glutamine-hydrolyzing) activity (MF); aspartate-ammonia ligase activity (MF); ATP binding (MF); cytoplasm (CC); asparagine biosynthetic process (BP); glutamine metabolic process (BP); cellular amino acid biosynthetic process (BP); cellular amino acid catabolic process (BP); amino acid binding (MF); identical protein binding (MF); L-asparagine biosynthetic process (BP)
XX999_00071	*bin3_2*	17298–17972	−	675	Putative transposon Tn552 DNA-invertase bin3	Recombinase activity (MF); DNA binding (MF); DNA integration (BP); transposition (BP)
XX999_00072	*ltrA_1*	18520–19686	+	1167	Group II intron-encoded protein LtrA	RNA-directed DNA polymerase activity (MF); endonuclease activity (MF); intron homing (BP); mRNA processing (BP)
XX999_00073	*hosA_1*	20060–20479	+	420	Transcriptional regulator HosA	DNA binding (MF); sequence-specific DNA binding transcription factor activity (MF); intracellular (CC); transcription, DNA-templated (BP); pathogenesis (BP)
XX999_00074	*XX999_00074*	20536–20991	+	456	hypothetical protein	—
XX999_00075	*XX999_00075*	20988–21206	+	219	hypothetical protein	—
XX999_00076	*XX999_00076*	21421–21912	+	492	hypothetical protein	—
XX999_00077	*XX999_00077*	22017–22805	+	789	flavodoxin	—
XX999_00078	*XX999_00078*	22823–23476	+	654	NmrA-like family protein	—
XX999_00079	*XX999_00079*	23512–24384	+	873	Alpha/beta hydrolase family protein	—
XX999_00080	*hsrA_2*	24631–24924	+	294	putative transport protein HsrA	Plasma membrane (CC); integral component of membrane (CC); transmembrane transport (BP)
XX999_00081	*efpA*	24921–25958	+	1038	putative MFS-type transporter EfpA	Plasma membrane (CC); integral component of membrane (CC); transmembrane transport (BP)
XX999_00082	*XX999_00082*	26043–26618	+	576	flavodoxin	—
XX999_00083	*glcU_1*	26631–27491	+	861	Glucose uptake protein GlcU	Plasma membrane (CC); rhamnose transmembrane transporter activity (MF); integral component of membrane (CC); sporulation resulting in formation of a cellular spore (BP)
XX999_00084	*yvgN_1*	27580–28431	+	852	Glyoxal reductase	Methylglyoxal reductase (NADPH-dependent) activity (MF)
XX999_00085	*gdhIV_1*	28460–29245	+	786	Glucose 1-dehydrogenase 4	Identical protein binding (MF); glucose 1-dehydrogenase [NAD(P)] activity (MF)
XX999_00086	*adhR_1*	29308–29703	+	396	HTH-type transcriptional regulator AdhR	DNA binding (MF); transcription, DNA-templated (BP); regulation of transcription, DNA-templated (BP)
XX999_00087	*XX999_00087*	29700–30434	+	735	putative oxidoreductase	Oxidoreductase activity (MF)
XX999_00088	*yhdN_1*	30459–31436	+	978	General stress protein 69	Oxidoreductase activity (MF)
XX999_00089	*XX999_00089*	31514–32101	+	588	Polysaccharide deacetylase	Hydrolase activity, acting on carbon-nitrogen (but not peptide) bonds (MF); polysaccharide binding (MF); endo-1,4-beta-xylanase activity (MF); xylan catabolic process (BP)
XX999_00090	*XX999_00090*	32681–33805	−	1125	hypothetical protein	—
XX999_00091	*XX999_00091*	33809–34024	−	216	hypothetical protein	—
XX999_00092	*topB_4*	34147–35697	−	1551	DNA topoisomerase 3	Magnesium ion binding (MF); DNA binding (MF); DNA topoisomerase type I activity (MF); DNA topological change (BP); DNA recombination (BP); chromosome separation (BP)
XX999_00093	*mobA_2*	35779–37842	−	2064	Mobilization protein A	Conjugation (BP); DNA binding (MF); DNA-directed RNA polymerase activity (MF); DNA topoisomerase type I activity (MF); cytoplasm (CC); metal ion binding (MF)
XX999_00094	*XX999_00094*	38344–38622	+	279	hypothetical protein	—

BP, biological process; CC, celular component; MF, molecular function.

**Table 5:** Genes determined in pLPE-4 plasmid of *Lactobacillus pentosus* MP-10 isolated from naturally fermented Aloreña table olives.

Gene ID	Gene	Position	Strand	Gen length (bp)	Protein description	GO terms	Similarity to proteins in *Lactobacillus*
XX999_00001	*XX999_00001*	99–314	−	216	hypothetical protein	—	100% identity in *L. plantarum* 90sk
XX999_00002	*XX999_00002*	435–803	−	369	DNA topoisomerase III	—	100% identity in *L*. paraplantarum DSM 10667
XX999_00003	*topB_1*	808–1116	−	309	DNA topoisomerase 3	Magnesium ion binding (MF); DNA binding (MF); DNA topoisomerase type I activity (MF); DNA topological change (biological_process); DNA recombination (BP); chromosome separation (BP)	100 identity in *L. paraplantarum* DSM 10667
XX999_00004	*topB_2*	1194–2567	−	1374	DNA topoisomerase 3	Magnesium ion binding (MF); DNA binding (MF); DNA topoisomerase type I activity (MF); DNA topological change (BP); DNA recombination (BP); chromosome separation (BP)	98% identity in *L. pentosus* IG1
XX999_00005	*XX999_00005*	2574–2984	−	411	hypothetical protein	—	100% identity in *L. plantarum* Lp1610
XX999_00006	*XX999_00006*	3000–3857	−	858	hypothetical protein	—	100% identity in *L. sakei* WiKim0063
XX999_00007	*XX999_00007*	3863–4237	−	375	hypothetical protein	—	100% identity in *L. pentosus*
XX999_00008	*traG_1*	4252–5796	−	1545	Conjugal transfer protein TraG	Conjugation (BP); DNA binding (MF); plasma membrane (CC); integral component of membrane (CC)	99% identity in *L. kefiranofaciens* subsp. *kefiranofaciens* DSM 5016
XX999_00009	*XX999_00009*	5840–6010	−	171	hypothetical protein	—	86% identity in *L. fermentum* MTCC 8711
XX999_00010	*XX999_00010*	6026–6496	−	471	hypothetical protein	—	97% identity in *L. paraplantarum*
XX999_00011	*XX999_00011*	6499–6867	−	369	hypothetical protein	—	91% identity in *L. plantarum*
XX999_00012	*XX999_00012*	6854–7471	−	618	hypothetical protein	—	99% identity in *L. brevis* DmCS_003
XX999_00013	*XX999_00013*	7486–8640	−	1155	Bacteriophage peptidoglycan hydrolase	—	99% identity in *L. brevis* KB290
XX999_00014	*XX999_00014*	8641–10059	−	1419	hypothetical protein	—	98% identity in *L. plantarum* Nizo2259
XX999_00015	*XX999_00015*	10052–12070	−	2019	AAA-like domain protein	—	99% identity in *L. parabuchneri* DSM 15352
XX999_00016	*XX999_00016*	12082–12741	−	660	hypothetical protein	—	100% identity in *L. plantarum* 2025
XX999_00017	*XX999_00017*	12710–13072	−	363	hypothetical protein	—	100% identity in *L. plantarum* CMPG5300
XX999_00018	*XX999_00018*	13093–13431	−	339	hypothetical protein	—	100% identity in *L. plantarum* Nizo2259
XX999_00019	*XX999_00019*	13433–14047	−	615	hypothetical protein	—	98% identity in *L. paracollinoides* DSM 15502
XX999_00020	*XX999_00020*	14061–14390	−	330	hypothetical protein	—	100% identity in *L. parakefiri* JCM 8573
XX999_00021	*mobA_1*	14473–16533	−	2061	Mobilization protein A	Conjugation (BP); DNA binding (MF); DNA-directed RNA polymerase activity (MF); DNA topoisomerase type I activity (MF); cytoplasm (CC); metal ion binding (MF)	100% identity in *L. pentosus*
XX999_00022	*XX999_00022*	16804–17013	+	210	hypothetical protein	—	100% identity in *L. pentosus*
XX999_00023	*XX999_00023*	17036–17314	+	279	hypothetical protein	—	100% identity in *L*.
XX999_00024	*XX999_00024*	17304–17984	−	681	Zeta toxin	—	100% identity in *L*.
XX999_00025	*XX999_00025*	17981–18172	−	192	hypothetical protein	—	100% identity in *L*.
XX999_00026	*XX999_00026*	18213–18494	−	282	RelB antitoxin	—	100% identity in *L*.
XX999_00027	*XX999_00027*	18899–19993	−	1095	hypothetical protein	—	100% identity in *L*.
XX999_00028	*XX999_00028*	20530–20805	−	276	hypothetical protein	—	100% identity in *L*.
XX999_00029	*XX999_00029*	20808–21857	−	1050	StbA protein	—	100% identity in *L*.
XX999_00030	*XX999_00030*	22519–23808	−	1290	hypothetical protein	—	100% identity in *L*.
XX999_00031	*dpnM*	23823–24686	−	864	Modification methylase DpnIIA	Nucleic acid binding (MF); site-specific DNA-methyltransferase (adenine-specific) activity (MF); DNA restriction-modification system (BP)	100% identity in *L*.
XX999_00032	*bin3_1*	24835–25416	−	582	Putative transposon Tn552 DNA-invertase bin3	Recombinase activity (MF); DNA binding (MF); DNA integration (BP); transposition (BP)	
XX999_00033	*XX999_00033*	25526–26605	−	1080	FRG domain protein	—	
XX999_00034	*hsrA_1*	27276–28667	+	1392	putative transport protein HsrA	Plasma membrane (CC); integral component of membrane (CC); transmembrane transport (BP)	
XX999_00035	*XX999_00035*	28667–29329	+	663	putative hydrolase	—	
XX999_00036	*XX999_00036*	29319–29720	+	402	hypothetical protein	—	
XX999_00037	*XX999_00037*	29820–30473	+	654	S-adenosyl-L-homocysteine hydrolase	Adenosylhomocysteinase activity (MF); cytoplasm (CC); one-carbon metabolic process (BP)	
XX999_00038	*XX999_00038*	31017–32141	−	1125	hypothetical protein	—	
XX999_00039	*XX999_00039*	32145–32360	−	216	hypothetical protein	—	
XX999_00040	*topB_3*	32482–34617	−	2136	DNA topoisomerase 3	Magnesium ion binding (MF); DNA binding (MF); DNA topoisomerase type I activity (MF); DNA topological change (BP); DNA recombination (BP); chromosome separation (BP)	
XX999_00041	*XX999_00041*	34624–35034	−	411	hypothetical protein	—	
XX999_00042	*XX999_00042*	35050–35907	−	858	hypothetical protein	—	
XX999_00043	*XX999_00043*	35913–36287	−	375	hypothetical protein	—	
XX999_00044	*traG_2*	36302–37846	−	1545	Conjugal transfer protein TraG	Conjugation (BP); DNA binding (MF); plasma membrane (CC); integral component of membrane (CC)	
XX999_00045	*XX999_00045*	37890–38060	−	171	hypothetical protein	—	
XX999_00046	*XX999_00046*	38076–38546	−	471	hypothetical protein	—	
XX999_00047	*XX999_00047*	38549–38917	−	369	hypothetical protein	—	
XX999_00048	*XX999_00048*	38904–39521	−	618	hypothetical protein	—	
XX999_00049	*XX999_00049*	39536–40690	−	1155	Bacteriophage peptidoglycan hydrolase	—	
XX999_00050	*XX999_00050*	40691–42004	−	1314	hypothetical protein	—	
XX999_00051	*XX999_00051*	42001–42108	−	108	hypothetical protein	—	
XX999_00052	*XX999_00052*	42101–43795	−	1695	AAA-like domain protein	Conjugation (BP); plasma membrane (CC)	

BP, biological process; CC, celular component; MF, molecular function.

**Table 6:** Genes determined in pLPE-5 plasmid of *Lactobacillus pentosus* MP-10 isolated from naturally fermented Aloreña table olives.

Gene ID	Gene	Position	Strand	Gen length (bp)	Protein description	GO terms	Similarity to proteins in *Lactobacillus*
XX999_03553	*XX999_03553*	763–1230	+	468	Hypothetical protein	—	95% identity in *L. plantarum* Nizo2814
XX999_03554	*XX999_03554*	1634–1915	+	282	Bifunctional antitoxin/transcriptional repressor RelB	DNA binding (MF); transcription, DNA-templated (BP); regulation of transcription, DNA-templated (BP)	99% identity in *L. plantarum* 16
XX999_03555	*XX999_03555*	1956–2147	+	192	Hypothetical protein	—	98% identity in *L. plantarum* Nizo2814
XX999_03556	*XX999_03556*	2224–2502	−	279	Hypothetical protein	—	100% identity in *L. farraginis* DSM 18382
XX999_03557	*XX999_03557*	2525–2734	−	210	Hypothetical protein	—	100% identity in *L. diolivorans* DSM 14421
XX999_03558	*mobA_6*	3004–5061	+	2058	Mobilization protein A	Conjugation (BP); DNA binding (MF); DNA-directed RNA polymerase activity (MF); DNA topoisomerase type I activity (MF); cytoplasm (CC); metal ion binding (MF)	99% identity in *L. plantarum* 2025
XX999_03559	*XX999_03559*	5168–5461	+	294	Hypothetical protein	—	100% identity in *L. plantarum* 2025
XX999_03560	*XX999_03560*	5501–6115	+	615	Hypothetical protein	—	100% identity in *L. plantarum*
XX999_03561	*XX999_03561*	6117–6455	+	339	Hypothetical protein	—	100% identity in *L. plantarum* 2025
XX999_03562	*XX999_03562*	6476–6838	+	363	Hypothetical protein	—	100% identity in *L. plantarum* IPLA88
XX999_03563	*XX999_03563*	6807–7466	+	660	Hypothetical protein	—	100% identity in *L. plantarum* CMPG5300
XX999_03564	*XX999_03564*	7478–9496	+	2019	AAA-like domain protein	Conjugation (BP); plasma membrane (CC)	99% identity in *L. plantarum*
XX999_03565	*XX999_03565*	9489–10904	+	1416	Hypothetical protein	—	99% identity in *L. plantarum* TMW 1.25 pL125–4 plasmid
XX999_03566	*XX999_03566*	10906–12075	+	1170	Bacteriophage peptidoglycan hydrolase	—	99% identity in *L. paraplantarum*
XX999_03567	*XX999_03567*	12090–12707	+	618	Hypothetical protein	—	100% identity in *L. plantarum* 2025
XX999_03568	*XX999_03568*	12685–13059	+	375	Hypothetical protein	—	99% identity in *L. plantarum* Nizo2814
XX999_03569	*XX999_03569*	13060–13518	+	459	Hypothetical protein	—	100% identity in *L. plantarum* 2025
XX999_03570	*traG_3*	13515–15044	+	1530	Conjugal transfer protein TraG	Conjugation (BP); DNA binding (MF); plasma membrane (CC); integral component of membrane (CC)	99% identity in *L. plantarum*
XX999_03571	*XX999_03571*	15057–15470	+	414	Hypothetical protein	—	99% identity in *L. plantarum* Nizo2814
XX999_03572	*XX999_03572*	15483–16352	+	870	Hypothetical protein	—	99% identity in *L. L. plantarum* Nizo2814
XX999_03573	*topB_5*	16369–18507	+	2139	DNA topoisomerase 3	Magnesium ion binding (MF); DNA binding (MF); DNA topoisomerase type I activity (MF); DNA topological change (BP); DNA recombination (BP); chromosome separation (BP)	98% identity in *L. plantarum* SRCM101060
XX999_03574	*XX999_03574*	18629–18844	+	216	Hypothetical protein	—	100% identity in *L. plantarum* Nizo1838
XX999_03575	*XX999_03575*	18848–19039	+	192	Hypothetical protein	—	83% identity in *L. collinoides* 237
XX999_03576	*XX999_03576*	18993–19250	+	258	Hypothetical protein	—	99% identity in *L. plantarum* Nizo2029
XX999_03577	*axeA1_3*	19530–20243	+	714	Acetylxylan esterase precursor	Xylan catabolic process (BP); acetylxylan esterase activity (MF)	100% identity in *L. plantarum* Nizo2029
XX999_03578	*XX999_03578*	20326–20994	−	669	Integrase core domain protein	—	100% identity in *L. tucceti* DSM 20183
XX999_03579	*XX999_03579*	20957–21163	−	207	Hypothetical protein	—	100% identity in *L. brevis* 47 f
XX999_03580	*ldh_7*	21343–22305	+	963	L-lactate dehydrogenase	L-lactate dehydrogenase activity (MF); cytoplasm (CC); glycolytic process (BP); cellular carbohydrate metabolic process (BP)	100% identity in *L. plantarum* Nizo2029
XX999_03581	*XX999_03581*	22735–22869	+	135	Hypothetical protein	—	95% identity in *L. backii* TMW 1.1991
XX999_03582	*XX999_03582*	23089–23295	−	207	Hypothetical protein	—	100% identity in *L. brevis* 47 f
XX999_03583	*ldh_8*	23475–24437	+	963	L-lactate dehydrogenase	L-lactate dehydrogenase activity (MF); cytoplasm (CC); glycolytic process (BP); cellular carbohydrate metabolic process (BP)	100% identity in *L. plantarum* Nizo2029
XX999_03584	*XX999_03584*	24867–25001	+	135	Hypothetical protein	—	95% identity in *L. backii* TMW 1.1991
XX999_03585	*XX999_03585*	24998–25501	−	504	Transposase DDE domain protein	—	100% identity in *L. plantarum* subsp. *plantarum* P-8
XX999_03586	*XX999_03586*	25459–25797	−	339	Hypothetical protein	—	98% identity in *L. plantarum* IPLA88
XX999_03587	*XX999_03587*	26046–26384	−	339	Hypothetical protein	—	100% identity in *L. plantarum* IPLA88
XX999_03588	*XX999_03588*	26499–27041	+	543	Hypothetical protein	—	100% identity in *L. pentosus*
XX999_03589	*XX999_03589*	27059–27859	+	801	Adenylate and Guanylate cyclase catalytic domain protein	—	100% identity in *L. pentosus*
XX999_03590	*ubiE_3*	27925–28629	+	705	Demethylmenaquinone methyltransferase	Methyltransferase activity (MF); menaquinone biosynthetic process (BP)	98% identity in *L. parakefiri* DSM 10551
XX999_03591	*XX999_03591*	28680–28781	+	102	Hypothetical protein	—	100% identity in *L. parakefiri* DSM 10551
XX999_03592	*XX999_03592*	28794–29021	+	228	ASCH domain protein	—	100% identity in *L. plantarum* Nizo2029
XX999_03593	*XX999_03593*	29337–30368	+	1032	Integrase core domain protein	—	99% identity in *L. plantarum* WCFS1
XX999_03594	*XX999_03594*	30453–31067	−	615	Cadmium resistance transporter	—	100% identity in *L. plantarum* SF2A35B
XX999_03595	*cadC*	31069–31437	−	369	putative positive regulator of cadmium resistance	Sequence-specific DNA binding transcription factor activity (MF); regulation of transcription, DNA-templated (BP)	100% identity in *L. plantarum* WCFS1
XX999_03596	*npr_2*	31787–33187	−	1401	NADH peroxidase	NADH peroxidase activity (MF); cell redox homeostasis (BP); flavin adenine dinucleotide binding (MF)	100% identity in *L. plantarum* Nizo1839
XX999_03597	*XX999_03597*	33361–33615	+	255	Hypothetical protein	—	—
XX999_03598	*XX999_03598*	33533–33862	−	330	Hypothetical protein	—	100% identity in *L. plantarum* WCFS1
XX999_03599	*arsB*	33879–35174	−	1296	Arsenical pump membrane protein	Plasma membrane (CC); arsenite transmembrane transporter activity (MF); arsenite transport (BP); integral component of membrane (CC); response to arsenic-containing substance (BP)	99% identity in *L. plantarum* SF2A35B
XX999_03600	*arsA*	35233–36963	−	1731	Arsenical pump-driving ATPase	ATP binding (MF); arsenite-transmembrane transporting ATPase activity (MF); detoxification of arsenic-containing substance (BP)	100% identity in *L. plantarum* WCFS1
XX999_03601	*arsD*	37047–37409	−	363	Arsenical resistance operon trans-acting repressor ArsD	DNA binding (MF); transcription, DNA-templated (BP); negative regulation of transcription, DNA-templated (BP); response to arsenic-containing substance (BP)	100% identity in *L. plantarum* WCFS1
XX999_03602	*arsR_3*	37396–37755	−	360	Arsenical resistance operon repressor	DNA binding (MF); sequence-specific DNA binding transcription factor activity (MF); intracellular (CC); transcription, DNA-templated (BP); response to arsenic-containing substance (BP)	100% identity in *L. plantarum* WCFS1
XX999_03603	*pinR*	39098–39679	+	582	Putative DNA-invertase from lambdoid prophage Rac	Recombinase activity (MF); DNA binding (MF); DNA integration (BP)	100% identity in *L. backii* TMW 1.1992
XX999_03604	*bin3_3*	40077–40709	+	633	Putative transposon Tn552 DNA-invertase bin3	Recombinase activity (MF); DNA binding (MF); DNA integration (BP); transposition (BP)	100% identity in *L. backii* TMW 1.1992
XX999_03605	*XX999_03605*	40806–41168	+	363	Hypothetical protein	—	98% identity in *L. backii* TMW 1.1992
XX999_03606	*XX999_03606*	41577–41990	−	414	Hypothetical protein	—	100% identity in *L. backii* TMW 1.1992
XX999_03607	*parA*	41987–42871	−	885	Chromosome partitioning protein ParA	ATP binding (MF); chromosome segregation (BP)	100% identity in *L. hokkaidonensis* JCM 18461
XX999_03608	*XX999_03608*	43459–44988	+	1530	Hypothetical protein	—	100% identity in *L. backii* TMW 1.1992
XX999_03609	*XX999_03609*	45128–45235	−	108	Hypothetical protein	—	—
XX999_03610	*XX999_03610*	45885–46475	−	591	Transposase, Mutator family	—	99% identity in *L. brevis* TMW 1.2113

BP, biological process; CC, celular component; MF, molecular function.

**Figure 1:**
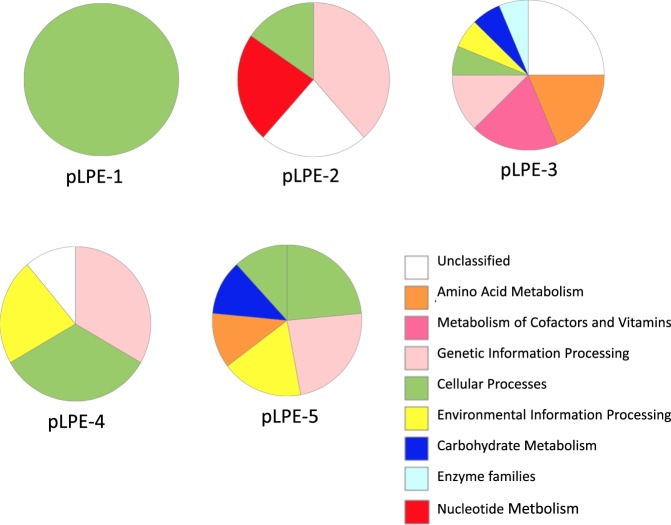
BlastKOALA results of functional gene-categories predicted in *Lactobacillus pentosus* MP-10 plasmids and their frequencies.

Figure 1 shows the frequency of KEGG functional annotations by BlastKOALA (KEGG tool; last updated March 4, 2016), which assigned plasmid genes to KEGG annotations corresponding to environmental information processing (pLPE-3, pLPE-4 and pLPE-5), genetic information processing (pLPE-2, pLPE-3, pLPE-4 and pLPE-5), carbohydrate metabolism (pLPE-3 and pLPE-5), amino acid metabolism (pLPE-3 and pLPE-5), cellular processes (pLPE-1, pLPE-2, pLPE-4 and pLPE-5), nucleotide metabolism (pLPE-2), metabolism of cofactors and vitamins (pLPE-3), and enzyme families (pLPE-3).

### *In silico* analysis of plasmid properties in *L. pentosus* MP-10

Analysis of the annotated CDs of each *L. pentosus* MP-10 plasmid revealed the presence of five genes involved in mobilization (*mobA* gene) distributed in all plasmids except the pLPE-2 plasmid (Tables 2–6). These genes are likely required for plasmid relaxation and mobilization by conjugative plasmids. Also, conjugation-related genes were found, e.g., *traG* in pLPE-4 (*traG_1* and *traG_2*) and pLPE-5 (*traG_3*) plasmids (Tables 5 and 6). A gene encoding for a bacteriophage peptidoglycan hydrolase that may have been involved in growth was found in pLPE-4 (*XX999_00013* and *XX999_00049*) and pLPE-5 (*XX999_03566*) plasmids (Tables 5 and 6).

The presence of mobile genetic elements in *L. pentosus* MP-10 plasmids (pLPE-2, pLPE-3, pLPE-4 and pLPE-5) was already reported by Abriouel *et al*.^2^ such as four putative transposon Tn552 DNA-invertase bin3 (four different genes of the same family), transposase DDE domain proteins (4 genes in pLPE 2 and pLPE5 plasmids), transposases of the mutator family (3 genes in pLPE2, pLPE3 and pLPE5 plasmids) and transposases (2 genes in pLPE-2 and pLPE-3 plasmids). Concerning integrases, one phage integrase family protein (pLPE-1 plasmid) and 9 integrase core domain proteins were detected in pLPE-2, pLPE-3 and pLPE-5 plasmids (Tables 3, 4 and 6). A gene *pinR* coding for DNA invertase from prophage was detected in pLPE-5 plasmid (Table 5).

Chloride- (*clcA_2*) and sodium- (*nhaS3_4*) transport genes harboured by pLPE-2 plasmid (Table 3) indicated that this plasmid was involved in salt-tolerance in brine solutions (plasmid curing experiments). Furthermore, a copy of the same genes *clcA_1, nhaS3_1, nhaS3_2* and *nhaS3_3* were also found in *L. pentosus* MP-10 chromosome with the aim to potentiate chloride and sodium tolerance in brines.

Genes related to carbohydrate metabolism were found on plasmids (besides on the chromosome) such as L-Lactate dehydrogenase in pLPE-5 plasmid (*ldh_7* and *ldh_8* genes) (Table 6), genes involved in glucose uptake and metabolism such as *glcU_1* and *gdhIV_1* genes in pLPE-3 plasmid (Table 4), and a gene involved in xylan catabolic process (*axeA1_3*) in pLPE-5 (Table 5). However, another gene involved in xylan catabolic process (*XX999_00089*) was only detected in pLPE-3 plasmid, but not on the chromosome (Table 4).

Toxins reported in *L. pentosus* MP-10 plasmids include mazF-toxin encoding gene (*XX999_03521*) detected in pLPE-1 plasmid, genes coding for Zeta toxins in pLPE-3 (*XX999_00053*) and pLPE-4 (*XX999_00024*) plasmids, and also for antitoxins such as RelB antitoxin (*XX999_00026*) in pLPE-4 plasmid and the bifunctional antitoxin/transcriptional repressor RelB in pLPE-5 plasmid (*XX999_03554*) (Tables 2, 4–6). MazF toxin is a desirable property in probiotic bacteria, and is only detected in plasmid DNA of *L. pentosus* MP-10, not in the chromosome. However, *L. pentosus* MP-10 has to protect itself from the MazF toxin without any MazE antitoxin. On the other hand, RelB antitoxins were found both on plasmids and on the chromosome; however, no RelB toxins were detected. Zeta toxins were detected both on the chromosome (one gene) and also on plasmid DNA (two genes); however, no antitoxin was detected.

Other coding genes for several functions, such as a serine-rich adhesin for platelets precursor (*sraP* gene), were detected in pLPE-2 plasmid but not on the chromosome (Table 3); genes coding for vitamin biosynthesis such as *panE_1* and *panE_2* genes coding for 2-dehydropantoate 2-reductase (biosynthesis of vitamin B5), a gene XX999_00068 coding for prephenate dehydratase (biosynthesis of phenylalanine, tyrosine and tryptophan), were detected on the pLPE-3 plasmid (Table 4) and also on the chromosome.

**Figure 2:**
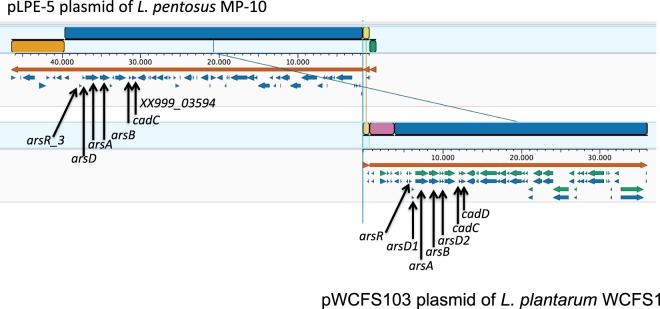
MAUVE visualization of the alignment of the pLPE-5 plasmid from *L*. *pentosus* MP-10 with the pWCFS103 plasmid from *L. plantarum* WCFS1. Arsenic- and cadmium-resistance genes are indicated.

Regarding their responses to stress, *in-silico* analysis of plasmid sequences revealed the presence of *yhdN_1* gene coding for a general stress protein 69 (in pLPE-3, Table 4) and several genes coding for metal tolerances, such as cadmium [cadmium resistance transporter (*XX999_03594*) and a putative positive regulator of cadmium resistance (*cadC*)] and two operons of arsenic resistance (in pLPE-5, Table 6). One *ars* operon consists of *arsR_3* (arsenical resistance operon repressor ArsR) and *arsB* [arsenical pump membrane protein (ArsB)], but lacks *arsC* gene (arsenate reductase ArsC); the other *ars* operon contains *arsA* [arsenical pump-driving ATPase (ArsA)] and *arsD* gene [arsenical resistance operon trans-acting represor (ArsD)] in pLPE-5 (Table 6). The synteny of arsenic-resistance genes was examined by comparing the annotated sequences of pLPE-5 and pWCFS103 plasmids (aligned by MAUVE algorithm) from *L*. *pentosus* MP-10 and *L. plantarum* WCFS1, respectively. Comparison revealed that the synteny of genes was similar (Fig. 2), being arsenic operons in pLPE-5 of *L. pentosus* MP-10 composing of two copies each gene: *arsB* [coding for trivalent As(III) efflux permease ArsB], *arsA* [coding for trivalent As(III)-stimulated ATPase ArsA], *arsD* [coding for trivalent As(III) metallochaperone ArsD] and *arsR_3* gene [a trivalent As(III)-responsive repressor (ArsR)]. On the other hand, *arsC* gene (*arsC2* coding for reductase ArsC), as a part of *ars* operon with *arsB* and *arsR* genes, was found in *L. pentosus* MP-10 chromosome, as well as two *arsR* gene copies (*arsR_1* and *arsR_2*).

### *In vitro* detection of functional properties in *L. pentosus* MP-10 plasmids

#### Effect of plasmid curing on growth of *L. pentosus* MP-10

The MIC of acridine orange (AO) was of 0.15 mg/ml; as such, we used 0.1 mg/ml as the sub-MIC for plasmid curing in this strain. After confirming *L. pentosus* MP-10 being cured of plasmids (data not shown), we compared the growth kinetics of uncured and cured *L. pentosus* MP-10C. The presence of plasmids did not affect the growth in MRS broth at 37 °C in any experimental conditions: presence/absence of 6.5% NaCl, different pH ranges (1.5 to 7.0), nor the presence of bile salts (1.8 or 3.6%) -no differences in 600 nm absorbances were detected over 24 h of incubation- (Figs S1–A,B, S2). In a similar manner, pH monitoring during their incubation also did not exhibit any significant differences between cured and uncured strains in regards to their acidification capacity (Fig. S1–C). Furthermore, no differences in the growth were detected between the cured and uncured *L. pentosus* MP-10 strains in the presence of xylan as the only carbohydrate source (Fig. S1–D). However, at high salt concentration of 8% usually found in brine, significant differences were detected between the cured and uncured *L. pentosus* MP-10 strains, with the uncured strain being the most tolerant (Fig. S1–E).

Table 7 shows that curing had no significant effect on the growth of uncured and cured *L. pentosus* MP-10 in the presence of phenolic compounds naturally present in the brines; both the cured and uncured strains tolerated more than 200 mg/ml of olive-leaf extract.

**Table 7:** Antibiotic and biodice susceptibility, and probiotic properties of cured and uncured *L. pentosus* MP-10 isolated from Aloreña Green table olives.

		MIC (µg/ml)	
		*L. pentosus* MP-10 (uncured)	*L. pentosus* MP-10C (cured)
Antibiotic	Amoxicillin	0.2	0.2
	Ampicillin	2	2
	Chloramphenicol	8	8
	Ciprofloxacin	16	16
	Clindamycin	**2**	**0.1**
	Gentamicin	0.1	0.1
	Kanamycin	4	4
	Streptomycin	4	4
	Teicoplanin	256	256
	Tetracycline	16	16
	Trimethoprim	0.125	0.125
	Trimethoprim/sulfometoxazole	0.125/2.38	0.125/2.38
	Vancomycin	2048	2048
Biocide	Benzalconium Chloride	2	2
	Triclosan	32	32
Phenolic componds		>2 × 10^5^	>2 × 10^5^
Probiotic properties	Auto-aggregation (%)	20.58 ± 2.54^a^	13.49 ± 0.54^a^
	Co-aggregation + *L. innocua* CECT 910 (%)	32.87 ± 2.14^a^	36.13 ± 2.33^a^
	Co-aggregation + *S. aureus* CECT 4468 (%)	28.61 ± 0.99^a^	28.69 ± 0.72^a^
	Co-aggregation + *E. coli* CCUG 47553 (%)	16.14 ± 2.09^a^	14.15 ± 3.24^a^
	Co-aggregation + *S*. Enteritidis UJ 3449 (%)	12.27 ± 1.50^a^	13.17 ± 2.87^a^
	Acid tolerance pH 2.0 (%)	100 ± 0.04^a^	100 ± 0.01^a^
	Acid tolerance pH 2.5 (%)	100 ± 0.03^a^	100 ± 0.02^a^
	Acid tolerance pH 3.0 (%)	100 ± 0.01^a^	100 ± 0.02^a^
	Bile tolerance at 1%	+	+
	Bile tolerance at 2%	+	+
	Bile tolerance at 3%	+	+
	Bile tolerance at 4%	+	+
	Mucin adhesion (%)	**55.93 ± 0.34** ^**a**^	**51.92 ± 1.06** ^**b**^

±SD, standard deviations of three independent experiments.

*Different lowercase letters represent significant differences according to 2-sided Tukey’s HSD between strains (*p* < 0.05). +, Presence of growth in MRS-agar with different concentrations of bile salts.

#### Effect of plasmid curing on metal tolerance

Plasmid annotations predicted gene clusters involved in arsenate- and/or arsenite-, and cadmium resistance. First, we precisely determined metal concentrations that inhibit the visible growth of the wildtype *L. pentosus* MP-10; results showed that this strain tolerated high concentrations of metals depending on the metal with 1 < MIC < 4096 μg/ml, and tolerances were observed to be in order Fe > [Al/Cu/Co] > Zn > Cd > Hg (Table 8). When we compared the uncured and the cured *L. pentosus* MP-10, we found that mercury and cadmium exibited different MICs among strains by 2–8 fold increase (Table 8) in those uncured; as such, plasmids have a key role in mercury and cadmium tolerances.

**Table 8:** Tolerance of cured and uncured *L. pentosus* MP-10 isolated from Aloreña Green table olives to heavy metals.

Metal	MIC (µg/ml)	
	*L. pentosus* MP-10 (uncured)	*L. pentosus* MP-10C (cured)
Mercury (Hg)	**2**	**1**
Cobalt (Co)	2048	2048
Copper (Cu)	2048	2048
Zinc (Zn)	1024	1024
Aluminium (Al)	2048	2048
Iron (Fe)	4096	4096
Cadmium (Cd)	**8**	**1**
	**% heavy metal removed**	
Mercury (Hg)	**81.74 ± 2.04** ^**a**^	**63.68 ± 1.09** ^**b**^
Cobalt (Co)	**10.65 ± 1.03** ^**a**^	**10.18 ± 0.67** ^**b**^
Copper (Cu)	**11.92 ± 0.45** ^**a**^	**7.41 ± 0.89** ^**b**^
Zinc (Zn)	**37.03 ± 1.02** ^**a**^	**34.73 ± 2.0** ^**b**^
Aluminium (Al)	**57.14 ± 0.99** ^**a**^	**49.92 ± 0.72** ^**b**^
Iron (Fe)	**21.04 ± 1.50** ^**a**^	**14.36 ± 0.78** ^**b**^
Cadmium (Cd)	**67.10 ± 0.88** ^**a**^	**55.40 ± 0.67** ^**b**^

±SD, standard deviations of three independent experiments.

*Different lowercase letters represent significant differences according to 2-sided Tukey’s HSD between strains (*p* < 0.05).

The removal of different metals was shown in Table 8, which demonstrated that *L. pentosus* MP-10 was able to remove different metals, thus exhibiting high removal capacity of mercury (81.74% ± 2.04), cadmium (67.10% ± 0.88) and aluminium (57.14% ± 0.99). However, the cured *L. pentosus* MP-10C demonstrated statistically significant reduced performance. Metal removal differences between the uncured and the cured *L. pentosus* MP-10 highlight the role of plasmids to remove iron, cadmium, aluminium, cobalt, copper, zinc and mercury (Table 8).

**Figure 3:**
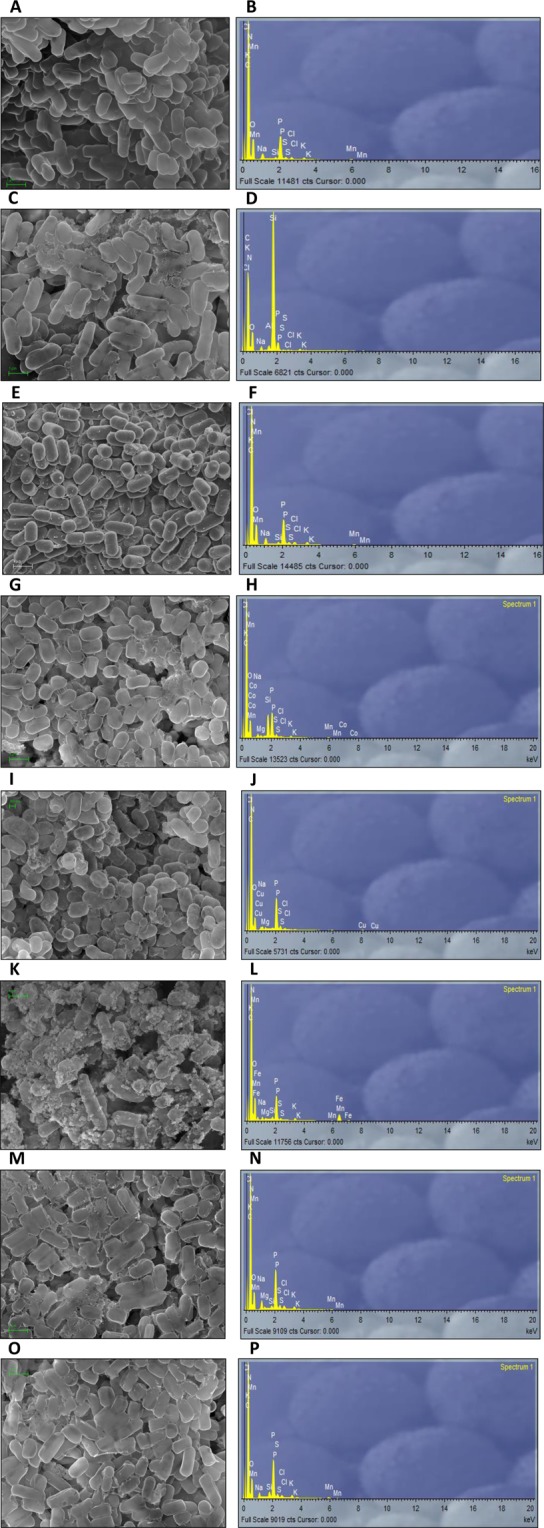
SEM (**A,C,E,G,I,K,M,O**) and EDX (**B,D,F,H,J,L,N,P**) analysis of uncured *L. pentosus* MP-10 without metal (**A,B**) and with Al (**C,D**), Cd (**E,F**), Co (**G,H**), Cu (**I,J**), Fe (**K,L**), Hg (**M,N**) and Zn (**O,P**).

To understand how *L. pentosus* MP-10 interact with selected metals, SEM analysis was performed and showed the biosorption potential of the uncured *L. pentosus* MP-10 (Fig. 3). The micrographs and EDX spectra obtained before and after the biosorption process showed clearly that the cell morphology of the uncured *L. pentosus* MP-10 changed and exhibited the presence of bright particles on the surface of the bacteria exposed to some metals. Regarding cadmium, mercury and zinc, we couldn´t detect these metals by EDX analysis. Furthermore, in the presence of either aluminium, cobalt, copper, mercury or zinc, higher potential for biofilm formation was observed. These results, confirmed by EDX analyses, support that these metals remained adsorbed entirely on the cell surface.

#### Effect of plasmid curing on antimicrobial resistance and probiotic features

We determined the MIC of different antibiotics and biocides between uncured and cured strains, and the results did not show any significant differences in response between both strains except for clindamycin, which exibited 20 fold increase in the MIC in the uncured *L. pentosus* MP-10. Thus, plasmids have no role in the suceptibility to the antibiotics and biocides tested, except clindamycin (Table 7).

Regarding the probiotic features, the uncured and the cured *L. pentosus* MP-10 had performed similarly in auto-aggregation and co-aggregation with all pathogens tested (Table 7), which suggest that plasmids had neither any role in auto-aggregation nor co-aggregation processes. Regarding acid and bile tolerance, no differences were detected between the uncured and the cured *L. pentosus* MP-10 (Table 7).

Adhesion to mucin was measured in both the uncured and the cured *L. pentosus* MP-10, and the results showed a statistically significant increase in adhesion capacity to mucin in the uncured *L. pentosus* MP-10 (Table 7).

## Discussion

Olive brine represents a stressful environment for the growth and survival of many bacteria due to the harsh conditions (i.e., high salt concentration, presence of phenolic compounds and low-nutrient availability), which provide selective pressures for the maintenance of LAB. As such, *L. plantarum* and *L. pentosus* have the genetic tools to survive and grow in the hostile olive-brine conditions^10^, and these genetic traits are widely distributed on both the chromosome and the plasmids, with several genes having multiple copies to enhance their adaptability and fitness in different ecological niches.

In this study, *L. pentosus* MP-10, isolated from Aloreña green table olives, harboured five plasmids with an average GC content (39.52–42.50%) slightly lower than the host chromosome (46.32%), this difference was less than 10% as reported by Nishida^25^ for the majority of plasmids. pLPE-5 had remarkably the lowest average GC content (39.52%) than the other four plasmids (pLPE-1, pLPE-2, pLPE-3 and pLPE-4), suggesting it is possibly a recent acquisition from another bacterium. *In-silico* analysis of plasmid sequences revealed the presence of genes involved in mobilization (*mobA*) and conjugation (*traG*) distributed in several plasmids, which suggest their role in gene mobilization and secretion using a type-IV secretion mechanism^26^. Furthermore, mobile genetic elements (e.g., transposon, transposase, integrase and invertase) were also found in several plasmids^2^ suggesting a frequent genetic diversification among the *L. pentosus* MP-10. Furthermore, bacteriophage peptidoglycan hydrolases were found in pLPE-4 and pLPE-5 plasmids; these lysozyme-like proteins may play a key role in *L. pentosus* MP-10 growth, its cell-wall structure, and immunomodulatory properties as reported by Rolain, *et al*.^27^.

Metabolic profile within *L. pentosus* MP-10 plasmids include carbohydrate enzymes such as L-lactate dehydrogenase, glucose uptake and metabolism and xylan catabolic enzymes. L-lactate dehydrogenase was codified by two genes (*ldh_7* and *ldh_8*) located on pLPE-5 plasmid; however, six L-lactate dehydrogenase (*ldh_1, ldh_2, ldh_3, ldh_4, ldh_5* and *ldh_6*) and four D-lactate dehydrogenase (*XX999_00315, XX999_00955, XX999_02047* and *XX999_02719*) coding genes were also present on the chromosome. Both enantiomers (L-lactate and D-lactate) are produced by *L. pentosus* MP-10 being D-and L-lactate dehydrogenases involved in the reversible metabolism of D- and L-lactate, respectively. This finding is of great interest suggesting that the use of *L. pentosus* MP-10 as a probiotic may help human to metabolise D-lactate obtained from exogenous sources (e.g., diet and the carbohydrate-fermenting bacteria normally present in the gastrointestinal tract) since mammalian cells lack sufficient D-lactate dehydrogenase required to utilise D-lactic acid—leading to chronic fatigue syndrome and D-lactic acidosis or D-lactate encephalopathy associated with short bowel syndrome^28–30^. Further, L-lactate dehydrogenase genes present on the plasmids may enhance their metabolic activity during the fermentation process to produce more L-lactate and energy. However, the presence of L-lactate dehydrogenase (*ldh_7* and *ldh_8*) coding genes on pLPE-5 plasmid did not enhance the acidification capacity, as results were similar after 8 and 24 h incubation in both cured and uncured *L. pentosus* MP-10, suggesting that these genes either have a minor role in lactate production or they are regulated. Further experiments, based on differential relative expression of *ldh* gene in both the cured and uncured *L. pentosus* MP-10 strains, revealed low expression level in the cured strain (Fig. S3), thus the low activity of lactate dehydrogenase gene in the cured strain is enough to give rise to a substantial lactate accumulation in the fermentation broth in a manner similar as the uncured strain. Regarding glucose uptake and metabolism, *glcU*_and *gdhIV* genes were over-expressed in the uncured *L. pentosus* MP-10 indicating the role of plasmid in this process (Fig. S3).

Among defense mechanisms found on plasmids, gene encoding the mazF toxin (pLPE-1), Zeta toxins (pLPE-3 and pLPE-4), and also antitoxins such as RelB antitoxin (pLPE-4) and the bifunctional antitoxin/transcriptional repressor RelB (pLPE-5) were detected in *L. pentosus* MP-10 plasmids. RelBE and MazEF are known as sequence-specific endo-ribonucleases that inhibit the global translations of cellular mRNAs^31^. MazF toxin is a desirable trait for probiotic bacteria, as its antimicrobial property inhibits several pathogens in foods and the gastrointestinal tract^32^. However, *L. pentosus* MP-10 must protect itself from the mazF toxin, as no MazE antitoxin was detected. Either their protection relies on other mechanisms because *mazF* is functional being only expressed in the uncured strain (Fig. S3). On the other hand, genes for RelB antitoxins were found both on plasmids and on the chromosome; however, no RelB-toxin genes were detected. So this antitoxin may contribute a greater defense against other bacteria possessing RelB toxins, possibly increasing its competitiveness and survival in several ecological niches including gastrointestinal tract. This feature was mainly linked to plasmid being *relB* antitoxin gene over-exopressed in the uncured strain (Fig. S3). Zeta toxins, which are kinases that kill bacteria through global inhibition of peptidoglycan synthesis^33^, are detected both on the chromosome and also on plasmid DNA of *L. pentosus* MP-10, however no antitoxin was detected. Overall, *L. pentosus* MP-10 harbored in their plasmids incomplete toxin-antitoxin systems unlike what occur naturally in bacterial genomes, since several toxins or antitoxins were detected without self protection.

Data obtained by *in-silico* analysis suggests that plasmid-borne genes mediate the persistence of lactobacilli under olive fermentation conditions and enhance their probiotic properties; however, this hypothesis requires further studies for confirmation. As such, plasmid curing experiments carried out with *L. pentosus* MP-10 showed several differences between the uncured and the cured strains regarding metal tolerances, removal and mucin adhesion. However, plasmid curing did not affect their tolerance to gastro-intestinal conditions (e.g., acids and bile salts); neither their ability to grow under determined conditions (i.e., different pH intervals, bile salts or sodium chloride of 6.5%) nor their colony morphology were changed after plasmid curing (data not shown). However, at high concentration of chloride of 8% (commonly added to brines), *L. pentosus* pLPE-2 plasmid plays a key role in salt tolerance. In this sense, the results suggest that the plasmids did not govern the fermentation of carbohydrates under these conditions, however different results were obtained by Adeyemo and Onilude^34^ which showed that plasmid curing had a significant negative effect on growth, physiological characteristics and colony morphology of *L. plantarum* isolated from fermented cereals. In this study, plasmids in *L. pentosus* MP-10 may confer a selective advantage, providing other physiological properties in certain environments such as gut and brines and thus allowing metal tolerance and removal, salt tolerance and adherence to mucin and thus their persistence in competitive ecological niches. Mucin adhesion declined in the cured *L. pentosus* MP-10 since a serine-rich adhesin for platelets precursor gene (*sraP*, detected in pLPE-2 plasmid) may be involved in mucin adhesion mechanisms similarly as reported by Hevia, *et al*.^34^ for an extracellular serine/threonine-rich protein as a novel aggregation-promoting factor with affinity to mucin in *Lactobacillus plantarum* NCIMB 8826. The role of *L. pentosus* MP-10 plasmids in mucin adhesion was confirmed by relative expression gene analysis as reported by Pérez Montoro *et al*.^35^, since *recA* and *pgm* genes considered as potential biomarkers of mucin adhesion were over-expressed in the uncured strain (Fig. S3). However, auto-aggregation and co-aggregation with some pathogens were not changed after plasmid curing of *L. pentosus* MP-10.

With respect to metals, which are considered non-biodegradable and non-thermodegradable and are of high concern in both developing and developed countries because of their impact on the environment and health (water and food), the wild strain *L. pentosus* MP-10 showed greater tolerance to their increased concentrations (MICs higher than 1 mg/ml, except for cadmium and mercury) of iron, cobalt, copper, aluminium and zinc. This suggests that high contamination of metals in the environment from natural and anthropogenic sources^36^ may be tolerable by the bacteria. The self-protective mechanisms displayed by *L. pentosus* MP-10 as a response to metals is promoted by their architecture (cell wall and membrane) and also by their resistance determinants located on the chromosome and the plasmids. Moreover, several chromosomally encoded cation transporters (e.g., encoded by *czcD* gene) have a predicted substrate range, including cadmium, cobalt and zinc; although the increased resistance towards different metals are displayed by plasmids (especially the pLPE-5 plasmid). Similar results were obtained by van Kranenburg *et al*.^22^, which reported that the plasmid-borne (pWCFS103) *cadC* gene coding for a transcription regulator of the cadmium operon was responsible of the increased resistance to cadmium in *L. plantarum* WCFS1. Furthermore, the synteny of *ars* genes in both *L. pentosus* MP-10 and *L. plantarum* WCFS1^22^ was similar suggesting their evolutionary relatedness. Arsenic and cadmium are among the most toxic elements widely ocurring in the environment, often a threat to food and water supply. Arsenic is known as a group A “known” carcinogen according to the United States Environmental Protection Agency (USEPA) and contributes to a range of other illnesses such as cardiovascular and peripheral vascular diseases, neurological disorders, diabetes mellitus and chronic kidney disease^37–39^. Detoxification of this metal was earlier established by bacteria. Thus, tolerance of *L. pentosus* MP-10 is necessary to prevent damage to their cells.

The ability of *L. pentosus* MP-10 to bind different metals was demonstrated by SEM and EDX analysis. This is of great importance with regards to their application as an adjunct to improve food safety and quality by bioquenching metals and probiotically reduce metal toxicity among human intestinal microbiota and thus protecting the host^40^. Also, we demonstrated that *L. pentosus* MP-10 contributed to metal removal, especially mercury and cadmium (81 and 67%, respectively).

Metal- and antibiotic-resistance genes often co-exist on the same plasmid, however in this case, we did not find any genes coding for clindamycin resistance on plasmids, which was the only antibiotic with different susceptibility after plasmid curing. Thus, clindamycin resistance in *L. pentosus* MP-10 may rely on other plasmid-associated genes that we could not deciphered yet.

## Conclusions

*In-silico* analysis of *L. pentosus* MP-10 plasmids suggests that plasmid-borne genes mediate the persistence of lactobacilli under olive-fermentation conditions and enhance their probiotic properties with genes encoding for carbohydrate metabolism, defense mechanisms, metal tolerance and mobilization increasing subsequently its competitiveness and survival in several ecological niches. Plasmid curing demonstrated the role of plasmids in the increased metal tolerance, and bioremoval of several metals (e.g., iron, aluminium, cobalt, copper, zinc, cadmium and mercury). This probiotic property by *L. pentosus* MP-10 should be exploited to detoxify metals in intestines; basically they could bioquench the metals in the gut thus reducing their toxic exposure to humans and animals, in the food matix and in environmental bioremediation.

## Materials and Methods

### Bacteria and growth conditions

*Lactobacillus pentosus* MP-10 isolated from naturally-fermented Aloreña green table olives^1^ were cultured in de Man Rogosa and Sharpe (MRS) broth (Fluka, Madrid, Spain) at 37 °C for 24 h. Pathogenic bacteria used in this study included *Listeria innocua* CECT 910, *Staphylococcus aureus* CECT 4468, *Escherichia coli* CCUG 47553, and *Salmonella* Enteritidis UJ3449, which were cultured in Tryptone Soya Broth (TSB; Fluka, Madrid, Spain) at 37 °C for 24 h. Cultures were maintained in 20% glycerol at −20 °C and −80 °C for short- and long-term storage, respectively.

### *In silico* analysis of *L. pentosus* MP-10 plasmid sequences

The genome sequence of *L. pentosus* MP-10 consisted of a single circular chromosome of 3,698,214 bp, with an estimated mol% G + C content of 46.32% and 5 plasmids ranging 29–46 kb (accession numbers FLYG01000001 to FLYG01000006) were annotated using the Prokka annotation pipeline, version 1.11 (Seemann, 2014) as previously reported by Abriouel, *et al*.^12^. The predicted CDSs of plasmids^2,12^ were annotated by using BLAST (Basic Local Alignment Search Tool) and the associated GO (Gene Ontology) terms were obtained by using Swiss-Prot database.

The general metabolic pathways of *L. pentosus* MP-10 plasmids were reconstructed using BlastKOALA (last updated March 4, 2016) as part of the KEGG (Kyoto Encyclopedia of Genes and Genome) tool in the pathway database (http://www.genome.jp/kegg/pathway.html) for annotating genomes; here, we used the annotated genes predicted in each *L. pentosus* MP-10 plasmid as the input query.

To evaluate the alignment and the synteny of genes between the *L*. *pentosus* MP-10 and *L. plantarum* WCFS1 plasmid data sets, comparison was done by using Mauve algorithm in Lasergene’s MegAlign Pro software (Lasergene 14).

### *In vitro* analysis of *L. pentosus* MP-10 plasmid properties

#### Plasmid curing

First, we determined the minimum inhibitory concentrations (MIC) of acridine orange (AO) to *L. pentosus* MP-10 using the broth micro-dilution method. Overnight cultures, grown in MRS broth at 37 °C for 24 h, were diluted 1/10 (v/v) in fresh MRS broth and 20 µl were added to each well of 96-well microtiter plates. 180 µl of MRS broth supplemented with AO at different concentrations (12.5–400 μg/ml) were then added to the wells and incubated at 37 °C under aerobic conditions for 24 h. Bacterial growth was evaluated by the presence of turbidity. MIC was defined as the lowest concentration of AO that inhibited visible growth. Each experiment was done in triplicate.

Plasmid curing (eliminating the plasmid from cells) of *L. pentosus* MP-10 was done as described by Adeyemo and Onilude^41^ with some modifications. Briefly, MRS broth (4 ml) supplemented with the sub-MIC of AO, as determined in this study, was inoculated with a selected colony of *L. pentosus* MP-10 grown onto MRS agar; then the cultures were incubated at 37 °C for 72 h. Serial dilutions of bacterial cultures in NaCl (0.85%) were plated onto MRS agar, and the resulting colonies, obtained after incubation for 48 h at 37 °C, were inoculated into MRS broth to obtain a pure culture. Cultures were maintained in 20% glycerol at −20 °C and −80 °C for short- and long-term storage, respectively.

To confirm that the resulting colonies were cured of plasmids, bacterial cultures (uncured and cured) were subjected to plasmid isolation as described by Abriouel, *et al*.^42^ and then visualized on 0.8% agarose gel electrophoresis (iNtRON Biotechnology) in 1xTBE (Tris-Boric acid-EDTA) buffer.

For additional confirmation, total genomic DNA (uncured and cured strains) was extracted using DNA Extraction Kit (Xtrem Biotech SL, Spain) according to the manufacturer´s instructions and tested for plasmid-borne genes. DNA quantification and quality assessment were carried out using a NanoDrop 2000 spectrophotometer (Thermo Scientific). DNAs were frozen at −20 °C until required and then subjected to PCR amplification of genes harboured by pLPE5, the biggest plasmid detected in *L. pentosus* MP-10. The PCR primers were designed in this study: Ars-pl5-F (5′-ATTATTTTGATCTCATTGATTTT-3′) and Ars-pl5-R (5′-TGAATAAACGAAACGGGAATGT-3′), yielding an amplicon of 570 bp. The 50 µl PCR mixture contained 20 ng of DNA, 0.5 μm of each primer (Ars-pl5-F and Ars-pl5-R), 200 μm of each deoxyribonucleoside triphosphate (Bioline), and 1 U of *Taq* DNA polymerase in 1X buffer according to the manufacturer’s instructions (Bioline). PCR was performed under the following conditions: one cycle at 95 °C for 3 min, 35 cycles at 95 °C for 30 s, 58 °C for 30 s, and 72 °C for 1 min and the final hold for 3 min at 72 °C. Analysis of PCR products was done by electrophoresis through a 1% agarose gel electrophoresis in 1xTBE (Tris-Boric acid-EDTA).

### Effect of plasmid curing on growth, safety and functional properties of *L. pentosus* MP-10

#### Growth properties

To test whether there is any differences in growth between the uncured and the cured *L. pentosus* MP-10 strains, MRS broth was inoculated (1% v/v) with overnight cultures of each strain and then incubated at 37 °C for 24 h. Growth rates (OD_600nm_) were measured each hour using Microtiter plate reader (iMark Microplate Absorbance Reader, Bio-Rad instrument). Additionally, we measured pH at different time intervals (following 0, 8 and 24 h of incubation at 37 °C).

To determine the effect of pH on the growth of both strains, MRS broth was adjusted to different pH ranges (1.5, 2.0, 2.5, 3.0, 3.5, 4.0, 4.5, 5.0, 5.5, 6.0, 6.5 and 7.0) with phosphate buffer, and they were inoculated (1% v/v) overnight cultures of both strains and then incubated at 37 °C for 24 h, as described above.

To test whether brine conditions had an effect on the growth of the plasmid-cured versus uncured *L. pentosus* MP-10 strains in MRS broths under the following experimental conditions: unsupplemented vs. those supplemented with either 6.5% (or high concentration of 8%) NaCl or phenolic compounds, or modified MRS broth (without glucose) added with xylan (5 g/l) were inoculated with both strains as described above. Phenolic compounds were obtained from freshly pulverized olive leaves using RETSCH laboratory ball mills (Retsh MM 400). The leaf extracts were resuspended in LSM broth, centrifuged and the resulting supernatant was filtered (0.45 μm) and added at different concentrations (0.780 to 200 mg/ml) to MRS broth. The cultures were incubated at 37 °C for 24 h and the OD_600nm_ was measured as described above.

In all cases, experiments were done in triplicate.

#### Evaluation of metal tolerance

The sensitivity of both *L. pentosus* strains (MP-10 and MP-10C (cured)) towards metals: cadmium (CdSO_4_·8/3H_2_O), cobalt (CoCl_2_), copper (CuCl_2_·2H_2_O), iron (FeSO_4_·7H_2_O), mercury (HgCl_2_), aluminium (Al_2_O_3_), or zinc (ZnCl_2_) was tested in LSM broth supplemented with 0 to 10 mg/ml of each metal and then inoculated with 2% (v/v) of an overnight culture of each strain. After 24 h of incubation at 37 °C, the MIC from each metal exposure was determined as described above, which corresponded to the lowest concentration that completely inhibited visible growth.

To analyse the removal of metals by cured and uncured *L. pentosus* MP-10, MRS broth supplemented with ½MIC of each metal was inoculated with 2% (v/v) of an overnight culture of each strain and then incubated 24 h at 37 °C. After incubation, the bacterial cells were removed by centrifugation and kept for the subsequent examination of metal sorption. The resulting supernatants were filter sterilized using a 0.22 μm filter (Millipore, Spain) and then used to check metal removal. MRS broth added either with different metals (with ½MIC) or not were used as positive and negative controls, respectively. The positive controls (MRS broth with individual metal added: Fe at 2 mg/ml; Al, Co and Cu at 1 mg/ml; Zn at 0.5 mg/ml; Cd at 4 μg/ml and 0.5 μg/ml; and Hg at 1 μg/ml and 0.5 μg/ml) were considered “100%” baselines to calculate relative metal removal rates (as a percentage).

Metal concentrations were measured using 7900 ICP-Mass Spectrometer (Agilent, USA) with graphite tube atomizer and autosampler, a superior matrix tolerance and advanced collision/reaction cell (CRC) technology to remove the polyatomic interferences that can affect some of the trace elements. The spectrometer software was Agilent ICP-MS MassHunter Work Station, which provides simple autotuning functions, and a Method Wizard automates the method setup process.

Biosorption of metals by *L. pentosus* MP-10 was further examined using scanning electron microscope (SEM) coupled with energy dispersive X-ray spectroscopy before and after metal uptake. For this, a drop of the bacterial pellet, which had been previously exposed to a metals (as previously described), were disposed into microporous capsules (ANAME, Spain), dried and then dehydrated in a series of 20, 40, 60, 80, and 100% ethanol solutions (15 min each) before suspension in acetone for 1 h. After this, the capsules were subjected to critical-point drying before examination by SEM (FESEM, MERLIN de Carl Zeiss, Oxford).

#### Safety and probiotic properties

To determine differences in antimicrobial (antibiotic and biocide) susceptibility of *L. pentosus* MP-10C versus wild strain, we determined the MIC of several antimicrobials following the method previously described by Casado Muñoz, *et al*.^42,43^ using LSM broth (Oxoid).

To determine if plasmids further play a role in several probiotic peroperties, we analyzed acid- and bile- tolerances, auto-aggregation, co-aggregation with pathogens (*L. innocua* CECT 910, *S. aureus* CECT 4468, *E. coli* CCUG 47553, and *S*. Enteritidis UJ3449) and mucin adhesion in both *L. pentosus* strains (MP-10 and MP-10C) according to the methods reported by Pérez Montoro *et al*.^35^.

#### Gene expression analysis

To analyse the role of plasmid in several metabolic and probiotic properties, both the uncured and cured L. pentosus strains were subjected to RNA extraction using Direct-zol™ RNA Miniprep (Zymo Research, California, USA) according to the manufacturer’s instructions. RNA quantification and quality assessment were carried out by using a NanoDrop 2000 spectrophotometer (Thermo Scientific). RNAs were adjusted to a concentration of 500 ng/ml and frozen at −80 °C until required for analysis.

The expression of selected genes (Table S1) was determined by quantitative, real-time PCR (qRT-PCR) using SensiFASTTM SYBR & Fluorescein One-Step Kit (BIOLINE) as reported in Pérez Montoro *et al*.^35^.

### Statistical analysis

All analyses were performed in triplicate. Statistical descriptors were calculated using Excel 2007 (Microsoft Corporation, Redmond, Washington, US), e.g., determining averages and standard deviations. Statistical comparison of growth and probiotic properties assays were conducted by analysis of variance (*ANOVA*) using Statgraphics Centurion XVI software (Statpoint Technologie, Warrenton, Virginia, US). The same software was used to perform Shapiro–Wilk and the Levene tests to check data normality and to perform 2-sided Tukey’s multiple contrast to determine the pair-wise differences between strains. Level of significance was set at *P* < 0.05.

## Supplementary information

Dataset 1
