# Supplementary material for: New insights into the role of plasmids from probiotic Lactobacillus pentosus MP-10 in Aloreña table olive brine fermentation
Source: Sci Rep. 2019 Jul 29;9:10938. doi: 10.1038/s41598-019-47384-1 (PMC6662855; doi:10.1038/s41598-019-47384-1)
Supplement: Supplementary file 1 — Dataset 1 [file 41598_2019_47384_MOESM1_ESM.pdf]

**New insights into the role of plasmids from probiotic *Lactobacillus pentosus* MP-10 in Aloreña table olive brine fermentation**

Hikmate Abriouel[1], Beatriz Pérez Montoro[1], Juan José de la Fuente Ordoñez[1], Leyre Lavilla Lerma[1], Charles W. Knapp[2], Nabil Benomar[1]

**Figure S1.** Growth kinetics of uncured and cured *L. pentosus* MP-10 in MRS broth (A), MRS broth supplemented with 6.5% NaCl (B) and pH monitoring after growth in MRS broth during different time intervals (0, 8 and 24 h) (C), modified MRS broth (without glucose) added with 5 g/l of xylan (D), and MRS broth supplemented with 8% NaCl (E). Data are expressed as mean values ± SD of three independent experiments.

**Figure S2.** Growth kinetics of uncured and cured *L. pentosus* MP-10 in MRS broth adjusted at different pH values (1.5-7.0) (A-L) and supplemented with bile salts (1.8 and 3.6%) (M-O). Data are expressed as mean values ± SD of three independent experiments.

**Figure S3.** The role of *L. pentosus* plasmids in metabolic and probiotic properties as revealed by the expression analysis of selected genes. Each bar represents mean value and standard deviation as error bar of three independent experiments. *significant differences between both the uncured and cured strains ($P < 0.05$).

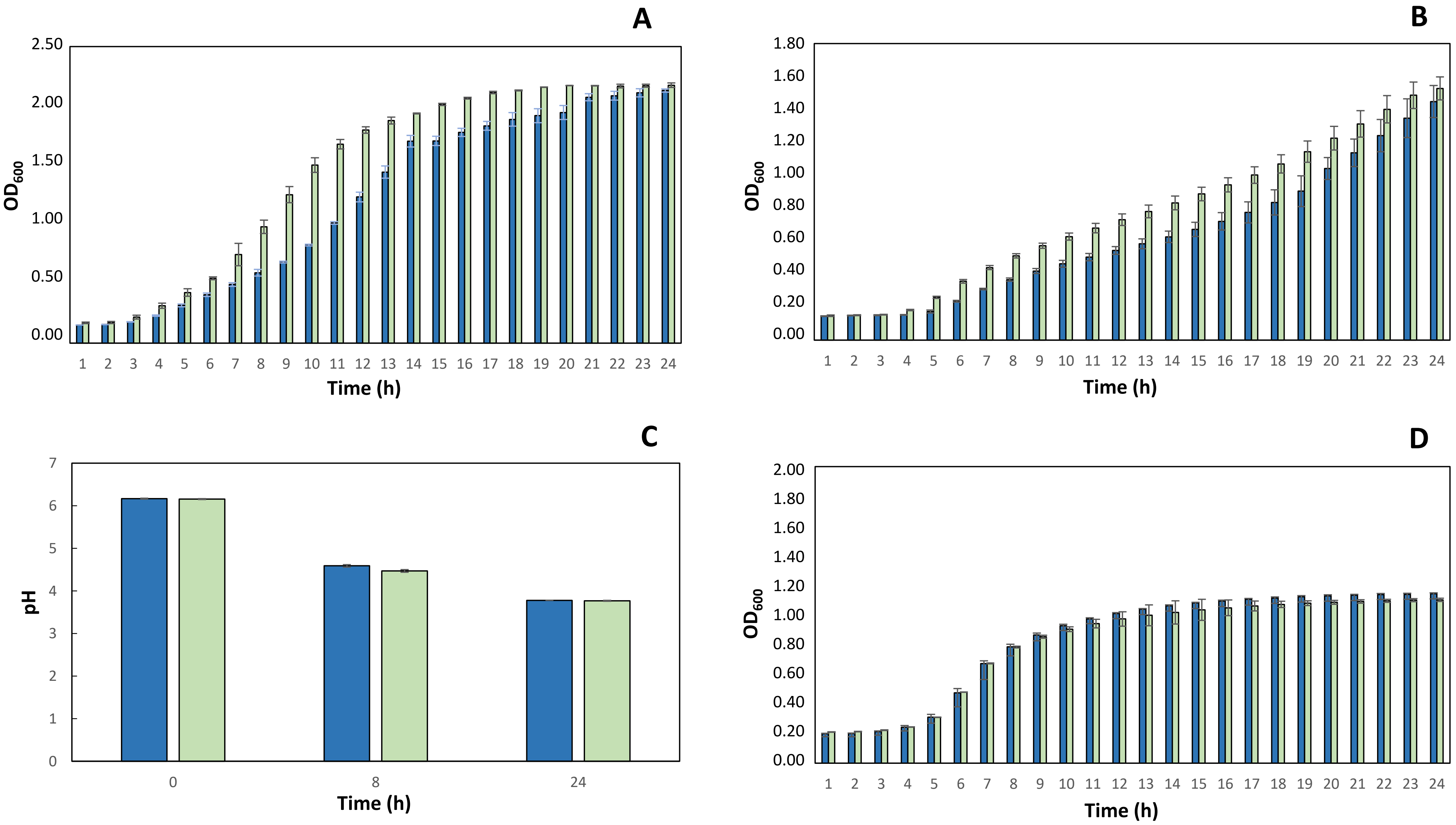

A
B
C
D
OD600
pH
Time (h)

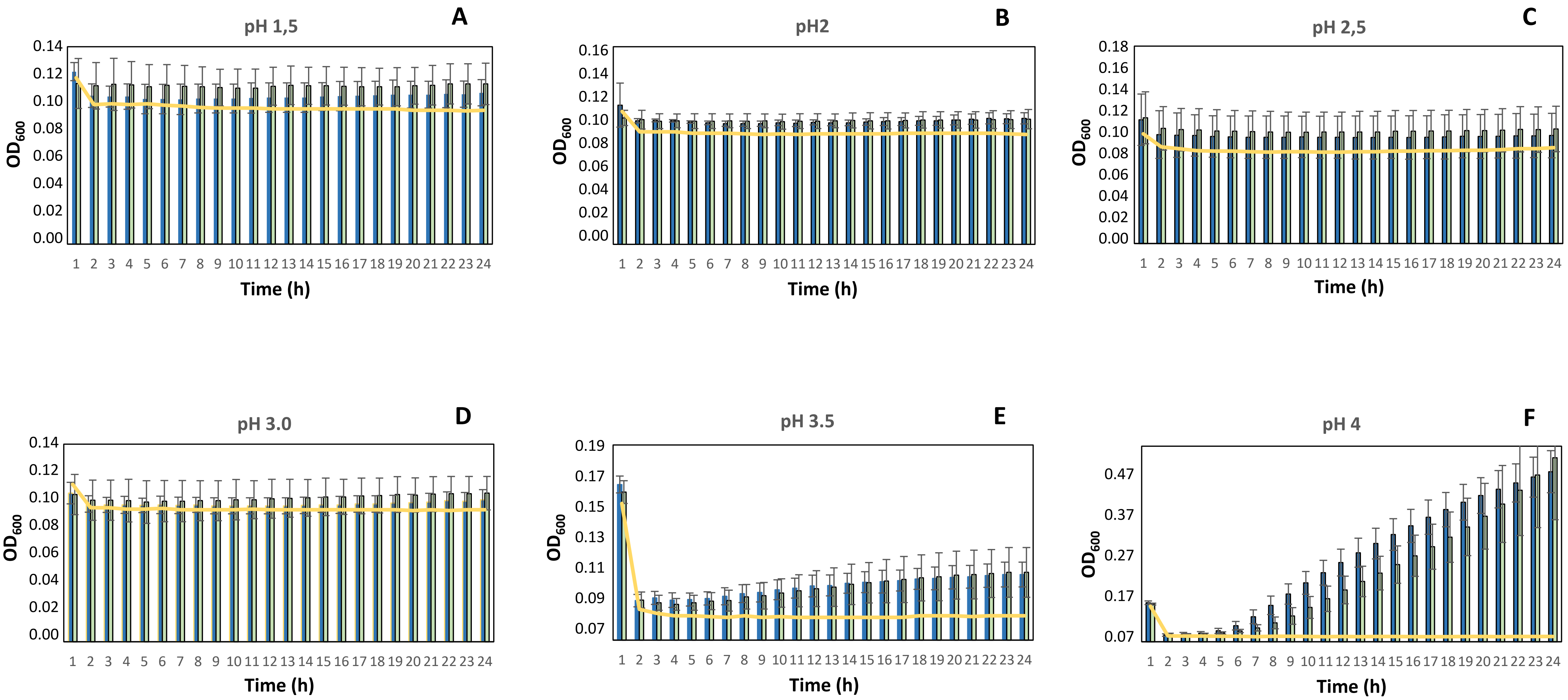
A
pH 1,5
B
pH2
C
pH 2,5
D
pH 3.0
E
pH 3.5
F
pH 4
OD600
Time (h)

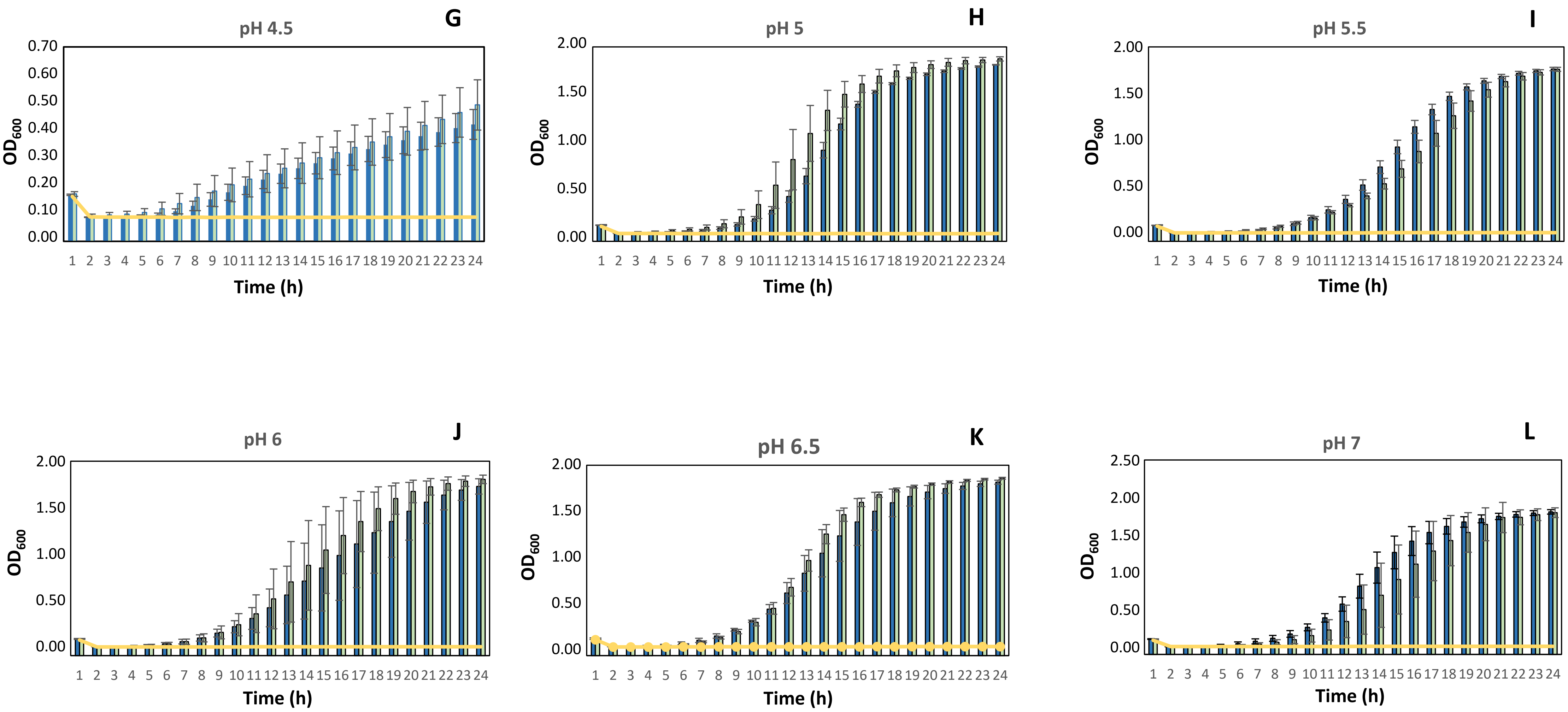

pH 4.5
G
0.70
0.60
0.50
0.40
0.30
0.20
0.10
0.00
OD600
1 2 3 4 5 6 7 8 9 10 11 12 13 14 15 16 17 18 19 20 21 22 23 24
Time (h)
pH 5
H
2.00
1.50
1.00
0.50
0.00
OD600
1 2 3 4 5 6 7 8 9 10 11 12 13 14 15 16 17 18 19 20 21 22 23 24
Time (h)
pH 5.5
I
2.00
1.50
1.00
0.50
0.00
OD600
1 2 3 4 5 6 7 8 9 10 11 12 13 14 15 16 17 18 19 20 21 22 23 24
Time (h)
pH 6
J
2.00
1.50
1.00
0.50
0.00
OD600
1 2 3 4 5 6 7 8 9 10 11 12 13 14 15 16 17 18 19 20 21 22 23 24
Time (h)
pH 6.5
K
2.00
1.50
1.00
0.50
0.00
OD600
1 2 3 4 5 6 7 8 9 10 11 12 13 14 15 16 17 18 19 20 21 22 23 24
Time (h)
pH 7
L
2.50
2.00
1.50
1.00
0.50
0.00
OD600
1 2 3 4 5 6 7 8 9 10 11 12 13 14 15 16 17 18 19 20 21 22 23 24
Time (h)

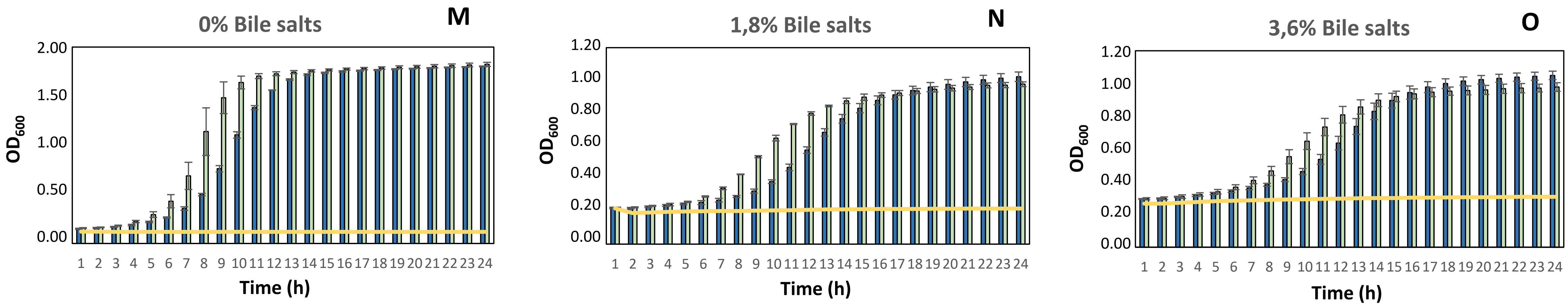


Figure S2

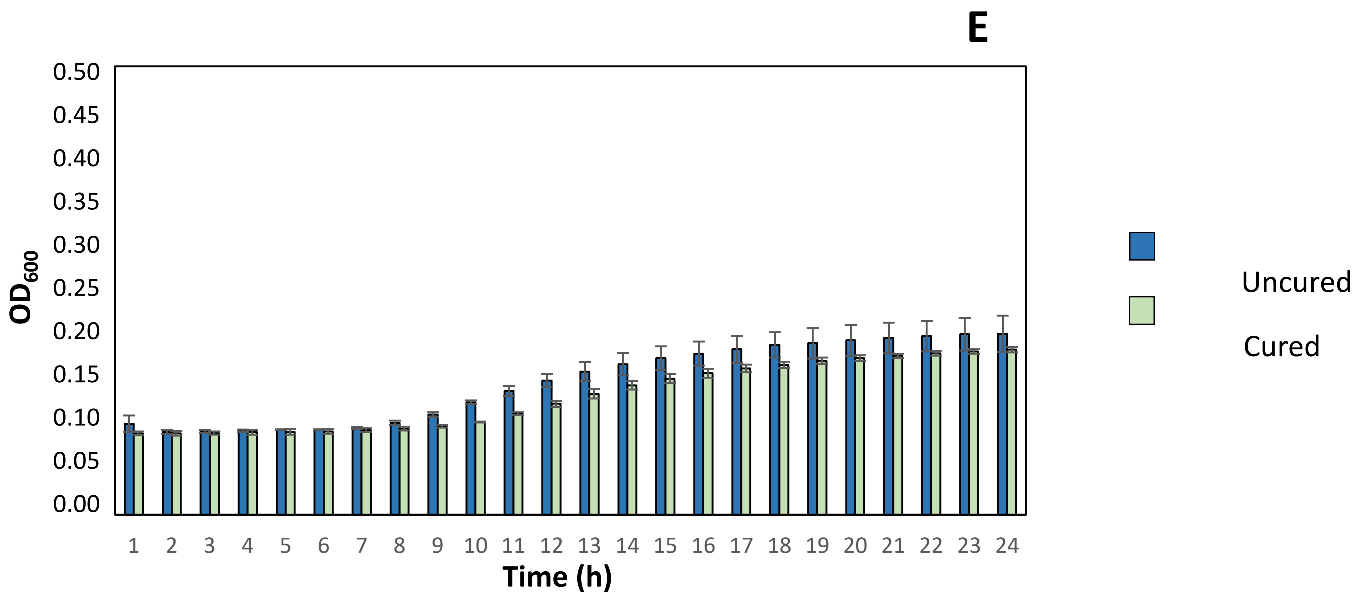
E
OD600
0.50
0.45
0.40
0.35
0.30
0.25
0.20
0.15
0.10
0.05
0.00
1 2 3 4 5 6 7 8 9 10 11 12 13 14 15 16 17 18 19 20 21 22 23 24
Time (h)
Uncured
Cured

**Figure S1**

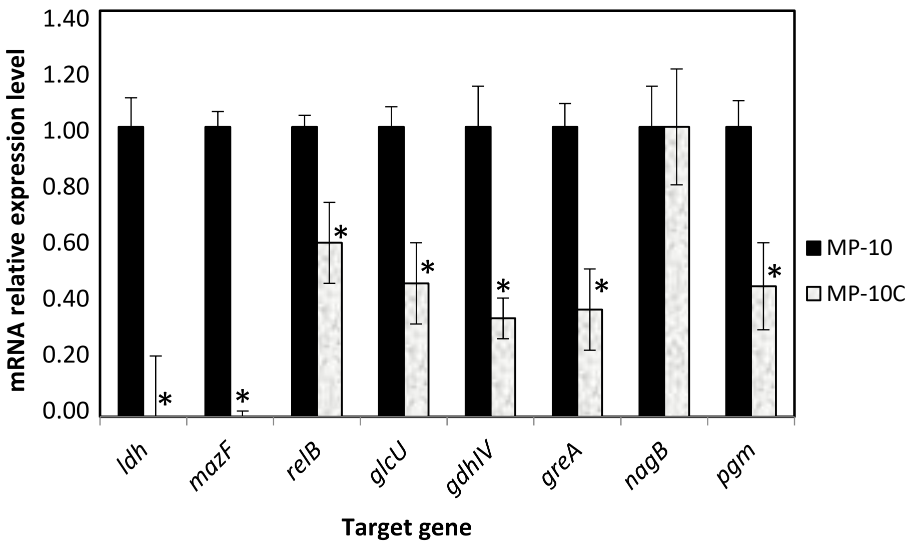

mRNA relative expression level
1.40
1.20
1.00
0.80
0.60
0.40
0.20
0.00
ldh
mazF
relB
glcU
gdhIV
greA
nagB
pgm
Target gene
MP-10
MP-10C


**Figure S3**

**Table 1.** Primers and PCR conditions used in this study.

| Primer | Sequence (5′-3′) | Annealing temperature (°C) | Reference |
|---|---|---|---|
| ***nagB-F*** | ATGAAAGTTATCGTAGTAAAG | 52 | Pérez Montoro et al.[35] |
| ***nagB-R*** | TGATTCCTTAAATGGCTTGT | 52 | Pérez Montoro et al.[35] |
| ***pgm-F*** | ATGGCGCAATTTTCAATTTACT | 54 | Pérez Montoro et al.[35] |
| ***pgm-R*** | AGCCGTAGAAGACTTCCCG | 54 | Pérez Montoro et al.[35] |
| ***greA-F*** | ATGGAACCAACTTTTAACAAA | 52 | Pérez Montoro et al.[35] |
| ***greA-R*** | TTGGACGATTTGGGCGTA | 52 | Pérez Montoro et al.[35] |
| ***pheS-21F*** | CAYCCNGCHCGYGAYATGC | 60 | Naser et al.[45] |
| ***pheS-23R*** | GGRTGRACCATVCCNGCHCC | 60 | Naser et al.[45] |
| ***ldh-F*** | ATCATCAAAAAGTTGTTTTAGTC | 58 | This study |
| ***ldh-R*** | AGCACCAGCTGTGATGACAA | 58 | This study |
| ***mazF-F*** | ATGACTTATTTGCCTAAGCAAA | 56 | This study |
| ***mazF-R*** | TCAAACGAATAAATCTGTGCAG | 56 | This study |

| | | | |
|---|---|---|---|
| ***relB-ant-F*** | ATGACTCGAGTATCAACTTCT | 58 | This study |
| ***relB-ant-R*** | ATCAGGAATGAGCCCTAATC | 58 | This study |
| ***glcU-F*** | ATGGCAATTATCTTAATGTTAT | 54 | This study |
| ***glcU-R*** | TTCCAATCAGCTGTAAACCA | 54 | This study |
| ***gdhIV-F*** | ATGCAGATTTAAACCAAAAAG | 54 | This study |
| ***gdhIV-R*** | ATCGCCAAAATTAGCTAATG | 54 | This study |
